# Analysis of structural variation and sex differentiation associated phylogenetic signals in newly sequenced *Rhodiola* chloroplast genomes using a batch processing pipeline

**DOI:** 10.3389/fpls.2026.1829588

**Published:** 2026-05-11

**Authors:** Erhuan Zang, Yanda Zhu, Dengxiu Ma, Hongmin Zou, Lingchao Zeng, Linchun Shi, Jinxin Liu

**Affiliations:** 1 School of Pharmacy, Minzu University of China, Beijing, China; 2 State Key Laboratory for Quality Ensurance and Sustainable Use of Dao-di Herbs, Institute of Medicinal Plant Development, Chinese Academy of Medical Sciences and Peking Union Medical College, Beijing, China; 3 Engineering Research Center of Chinese Medicine Resource, Ministry of Education, Beijing, China

**Keywords:** batch processing, chloroplast genome, nucleotide diversity, phylogenetic analysis, *Rhodiola*, sex differentiation, structural variation

## Abstract

**Introduction:**

*Rhodiola* is one of the few genera in Crassulaceae that includes both dioecious and hermaphroditic species. However, previous studies have mainly relied on representative individuals or limited plastid fragments, which may not fully resolve evolutionary relationships within the genus, especially lineage divergence associated with sexual-system differentiation.

**Methods:**

In this study, we analyzed the complete chloroplast genomes of 89 newly sequenced samples representing 35 *Rhodiola* species. A batch-processing pipeline was used to integrate raw-read processing, automated chloroplast genome assembly and annotation, and downstream comparative genomic and phylogenetic analyses.

**Results:**

The chloroplast genomes of *Rhodiola* were highly conserved in overall structure, gene content, and codon usage, while showing moderate sequence variation in several coding genes and intergenic regions. Highly variable regions included the coding genes *ycf1, matK, rpoC2*, and *rpoB*, as well as the intergenic regions *trnR*-UCU–*atpA*, *trnH*-GUG–*psbA*, and *rpl14–rpl16*. A rare 108-bp expansion in the *ndhA–ndhH* intergenic region was detected exclusively in *R. discolor*. Phylogenetic analysis supported two major clades broadly corresponding to hermaphroditic and dioecious lineages. However, the placements of *R. sachalinensis, R. wallichiana*, and *R. wallichiana* var. *cholaensis* indicate a more complex evolutionary history, potentially involving parallel evolution of sexual systems or transitional lineages. Lineage-specific polymorphisms in *rps15*, *ndhI*, *rps3*, and *rpl20* were further identified as candidate chloroplast genome markers associated with maternal lineage divergence and sexual-system evolution.

**Discussion:**

These findings demonstrate that complete chloroplast genome data provide higher resolution for understanding evolutionary relationships within *Rhodiola*. The identified highly variable regions and lineage-specific polymorphisms offer useful molecular markers for species identification and provide new insights into chloroplast genome evolution and reproductive-trait divergence in *Rhodiola*.

## 1 Introduction

*Rhodiola* L., a large genus in the family Crassulaceae, comprises approximately 90 species worldwide. It is mainly distributed across the Qinghai–Tibet Plateau and adjacent regions, including the Himalayas, the Hengduan Mountains, and the mountains of Central Asia, making it an important component of alpine plant diversity in this region. As a traditional and highly valued medicinal herb with a long history of use, *Rhodiola* exhibits a wide range of pharmacological activities, including anti-fatigue, anti-hypoxia, and immunomodulatory effects (Cunningham et al., 2020; Zhu et al., 2022). Among its species, *R. crenulata* and *R. sacra* are included in the Chinese Pharmacopoeia, whereas *R. rosea* is listed in the United States Pharmacopeia. In addition, *R. sachalinensis*, *R. quadrifida*, *R. algida*, *R. wallichiana*, *R. dumulosa*, and *R. kirilowii* are included in regional standards of China. Their major bioactive constituents, such as salidroside and other secondary metabolites, have attracted considerable research attention (Lai et al., 2024). However, because of overexploitation and habitat degradation, all *Rhodiola* species have been included in Appendix II of CITES, highlighting the urgent need for systematic genetic studies to support effective conservation and sustainable utilization.

Species of *Rhodiola* are mostly perennial herbs that inhabit harsh alpine environments at elevations of 3,500–5,000 m, including alpine meadows, scree slopes, and rock crevices. They exhibit substantial morphological diversity, particularly in rhizome form, flowering stem architecture, inflorescence morphology, and cauline leaf shape. This morphological complexity is closely associated with the rapid radiation history of the genus and has contributed to long-standing taxonomic uncertainty within *Rhodiola* (Huang et al., 2025). Notably, *Rhodiola* is one of the few genera in Crassulaceae that includes both dioecious and hermaphroditic species. The occurrence of dioecy in these alpine plants has been proposed as an adaptive response to high-altitude environmental pressures, such as limited pollinator availability and the need for optimized resource allocation (Zhang et al., 2014a).

As semi-autonomous organelles in plants, chloroplasts possess genomes that are structurally conserved, maternally inherited, and subject to relatively moderate rates of evolution. These characteristics make plastomes valuable molecular resources for resolving phylogenetic relationships, tracing species evolutionary history, and examining structural variation, including changes in genome size, inverted repeat (IR) boundary dynamics, and repetitive sequence distribution, some of which may be associated with adaptive evolution and trait divergence (Zhang et al., 2014b). In recent years, the research group led by Jianqiang Zhang has made important progress in elucidating the phylogeny of *Rhodiola*. Early studies based on a limited number of chloroplast fragments and nuclear ITS sequences were insufficient to fully resolve the complex phylogenetic relationships within the genus (Zhang et al., 2014c). Using plastome data from 23 representative species, this group subsequently reconstructed a robust phylogenetic framework for *Rhodiola* and identified two major clades that were strongly associated with sexual systems: one clade composed predominantly of hermaphroditic species, with the exception of *R. stapfii* and *R. integrifolia*, and the other consisting entirely of dioecious species. By integrating biogeographic and trait evolution analyses, they further demonstrated the parallel evolution of dioecy within the genus (Zhao et al., 2021). In addition, they detected three genes with positively selected sites (*rpl16*, *ndhA*, and *ndhH*) and one gene with an accelerated evolutionary rate (*psaA*), suggesting that plastome evolution may be involved in the adaptation of *Rhodiola* to high-altitude environments (Zhao et al., 2020). Subsequent studies based on nuclear genomic data further revealed extensive cytonuclear discordance and reticulate evolutionary processes during the diversification of *Rhodiola* (Ren et al., 2023; Huang et al., 2025). Collectively, these studies have demonstrated the utility of chloroplast genome data in improving phylogenetic resolution in recently radiated plant lineages. Nevertheless, previous studies have largely relied on representative individuals or publicly available sequence data. Sampling only one or a few individuals per species may fail to capture the full extent of intraspecific variation and may therefore obscure the true evolutionary relationships among species, especially for complex traits such as sex differentiation that may be associated with specific lineages or populations. A more robust understanding of these relationships therefore requires chloroplast genome data at a broader, population-level sampling scale (Kan et al., 2024).

Building on previous work (Liu et al., 2024), the present study substantially expands sampling by including multiple individuals per species. Our samples cover the major distribution regions of the genus, including the Qinghai–Tibet Plateau and adjacent areas, as well as Hebei, Sichuan, Yunnan, and Xinjiang, and represent the principal subgenera, sections, and series within *Rhodiola*. Using a batch processing pipeline, we assembled and analyzed 89 newly sequenced *Rhodiola* chloroplast genomes to systematically characterize structural variation in the genus, including variation in genome size, IR boundary dynamics, and repetitive sequence distribution. In addition, through phylogenetic analyses, we sought to reassess, at a population scale, how sex differentiation is distributed across phylogenetic lineages. This study is expected to provide a stronger population-level genomic framework for understanding the evolutionary patterns of sex differentiation during the rapid radiation of this alpine genus on the Qinghai–Tibet Plateau.

## 2 Materials and methods

### 2.1 Sample collection

A total of 89 samples representing 35 *Rhodiola* species were collected for this study (**Supplementary Table 1**). The sampling range covered the Qinghai–Tibet Plateau and adjacent regions, as well as the Taibai Mountains, Changbai Mountains, Bashang Plateau, and Helan Mountains. Species identification based on morphological characteristics was performed by Yulin Lin, Yaodong Qi, Xinlei Zhao, and Jinxin Liu from the Institute of Medicinal Plant Development, Chinese Academy of Medical Sciences, together with Jun Zhang from Yunnan Minzu University. Concurrently, molecular identification of *Rhodiola* samples was conducted using the *Rhodiola*-IDE identification software previously developed by our group, combined with DNA barcoding technology (Liu et al., 2025). All voucher specimens were deposited in the herbarium of the Institute of Medicinal Plant Development, Chinese Academy of Medical Sciences.

### 2.2 DNA sequencing, batch assembly, and annotation

Total genomic DNA was extracted using a modified CTAB method. Briefly, genomic DNA was isolated from 20–30 mg of dried tissue or 50–60 mg of frozen tissue using extraction buffer containing 2% CTAB, 1.4 M NaCl, and 0.3% β-mercaptoethanol, followed by incubation at 65 °C for 2 h. After quality assessment, library construction and paired-end sequencing (PE150) were performed on the Illumina NovaSeq platform by Berry Genomics Co., Ltd. (Beijing, China). After obtaining raw sequencing data, a batch processing pipeline based on custom scripts was used for chloroplast genome assembly and annotation. First, the raw sequencing data were standardized and renamed according to species names and collection site information, and datasets with identical names were merged. Quality control, filtering, and adapter trimming were then performed using Trimmomatic v0.39 (Bolger et al., 2014). Clean reads were subsequently used for batch assembly of chloroplast genomes with GetOrganelle v1.7.7.1 (Jin et al., 2020). Because the assembly results contained two possible genome orientations, a previously annotated *R. rosea* chloroplast genome GenBank file (accession number: PP262137) was used as the reference to determine the forward-oriented sequence. For samples in which GetOrganelle failed to generate complete genomes, the de Bruijn graphs of the assembly results were manually inspected and adjusted in Bandage v0.8.1 (Wick et al., 2015), and the corrected genome sequences were then exported. Finally, the starting positions of all genomes were adjusted according to the reference GenBank file, and genome annotation was performed using the command-line version of CPGAVAS2 (Shi et al., 2019), followed by manual correction.

### 2.3 Genome structure and comparative analysis

In addition to genome assembly and annotation, we developed a batch-processing pipeline for downstream chloroplast genome analyses. For structural and comparative analyses, we focused primarily on IR boundary regions, repetitive sequences, and basic chloroplast genome features. To identify IR regions, self-to-self BLASTn v2.16.0+ searches (Camacho et al., 2009) were performed using the parameters -outfmt 6 and -evalue 1e-5, with candidate inverted repeat lengths constrained to 18,000-32,000 bp. Based on the identified IR coordinates and the corresponding GenBank files, information on genes spanning or adjacent to the IR boundaries was extracted. IR boundary structures were then visualized using IRscope v3.1 (Amiryousefi et al., 2018).

We also developed custom scripts for batch calculation of relative synonymous codon usage (RSCU), and the resulting data were visualized as heatmaps using TBtools v2.2.2 (Chen et al., 2023). For repetitive-sequence analysis, multiple command-line tools were integrated into the custom pipeline, including MISA v2.1 (Beier et al., 2017), Tandem Repeats Finder v4.09.1 (Benson, 1999), and vmatch v2.3.1. MISA was used to detect simple sequence repeats (SSRs), with the minimum repeat thresholds set to 10 for mononucleotide repeats, 5 for dinucleotide repeats, 4 for trinucleotide repeats, and 3 for tetra-, penta-, and hexanucleotide repeats. Tandem Repeats Finder was used to identify tandem repeats with the parameters recommended in the official documentation (2 7 7 80 10 50 500 -f -d -m), whereas vmatch was used to identify dispersed repeats, including forward and palindromic repeats. In addition, two custom scripts were developed to summarize basic genomic features. One script was used to calculate general chloroplast genome statistics, including total genome size, GC content, lengths of the quadripartite regions (LSC, SSC, and the two IRs), and codon-usage information. The other script was designed specifically to calculate RSCU values.

### 2.4 Nucleotide diversity and identification of variable regions

To assess the extent of sequence variation within *Rhodiola* chloroplast genomes and to identify potential hypervariable regions as candidate molecular markers, we conducted nucleotide diversity analyses across all samples. For batch processing, a custom script was developed to extract coding sequences (CDSs) and intergenic spacer (IGS) regions from GenBank files. Region names were then standardized, and a reference genome was used to determine the correspondence of IGS regions among samples. After multiple sequence alignment with MAFFT v2.20 (Katoh and Standley, 2013), nucleotide diversity (Pi) was calculated for each aligned region.

The number of SNP sites for each aligned region was recorded in MEGA using the Sequence Data Explorer (Kumar et al., 2018), where SNPs were defined as variable nucleotide positions in the multiple sequence alignment. Thus, the SNP count represented the total number of polymorphic sites in a region, irrespective of their frequencies among samples. In contrast, Pi was calculated with a self-written script following the definition of Nei and Li (Nei and Li, 1979) as the average number of nucleotide differences per site among all pairwise sequence comparisons. Therefore, unlike SNP count, Pi reflects not only the number of variable sites but also the frequency distribution of those variants across samples. Regions containing a small number of lineage-specific but high-frequency substitutions may consequently show relatively high Pi values despite having relatively few SNP sites.

### 2.5 Phylogenetic analysis

To reconstruct the phylogenetic relationships, homologous chloroplast CDSs were extracted from the 89 *Rhodiola* samples and three outgroup species (accession numbers: MN064718.1, NC_023085.1, and PP234493.1) using a custom script. The extracted CDSs were aligned using MAFFT v2.20, and all aligned CDSs from the same sample were concatenated into a single FASTA matrix. The concatenated dataset was imported into MEGA12 for phylogenetic reconstruction using the Maximum Likelihood (ML) method under the GTR+GAMMA+I substitution model, with branch support evaluated by 1000 bootstrap replicates. Sex phenotype information for each sample, including dioecy and hermaphroditism, was subsequently mapped onto the phylogenetic tree to visualize the relationship between sexual-system differentiation and phylogenetic lineages.

## 3 Results

### 3.1 Large-scale chloroplast genome assembly, annotation, and data processing

**Figure 1:**
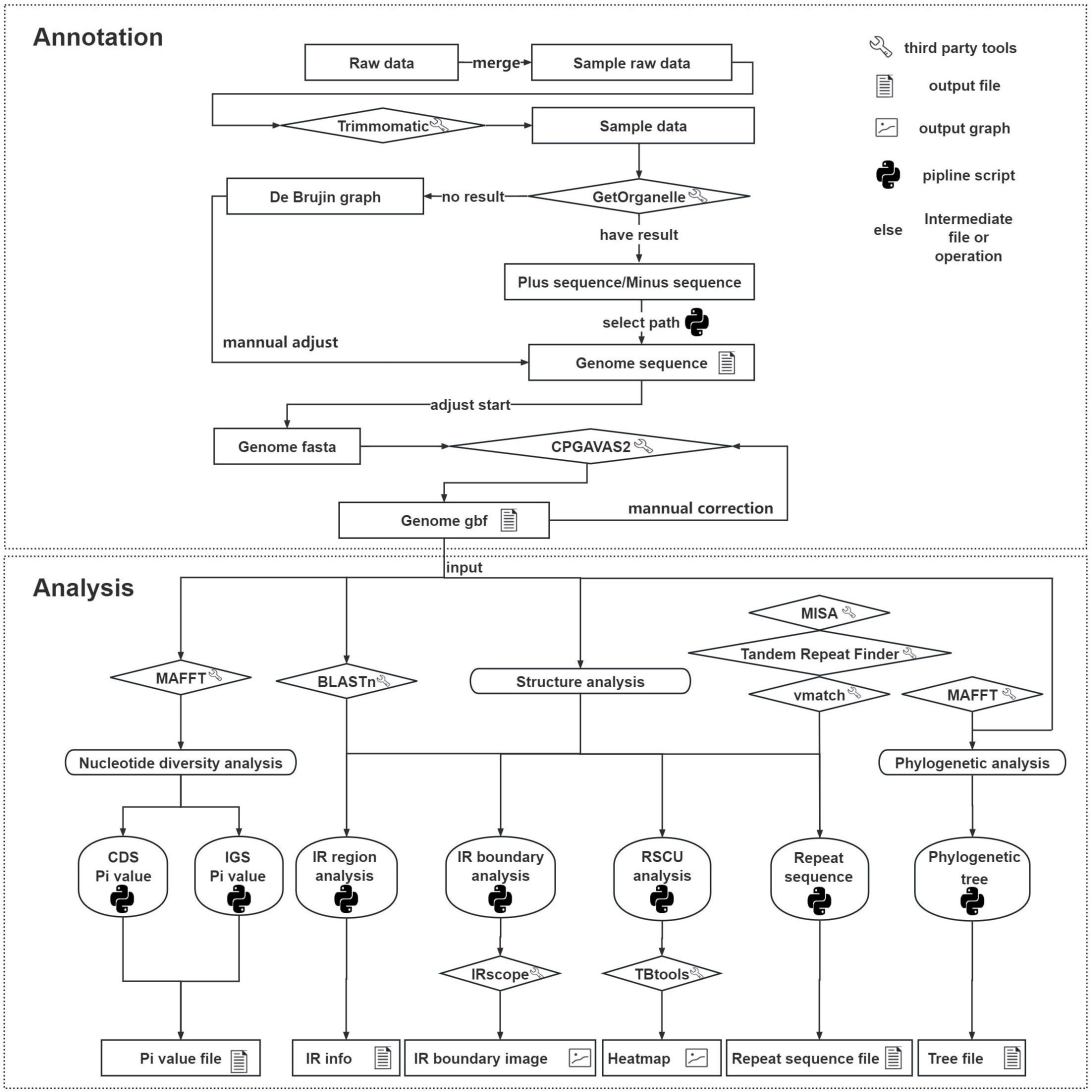
Pipeline for large-scale analysis of chloroplast genomes, including two main sections: assembly/annotation and data analysis. Legend is shown at the top right.

Traditional chloroplast genome analysis generally relies on a standardized workflow designed for only a small number of genomes. Because different analytical steps often require switching among multiple software tools, and some programs do not support batch processing, conventional workflows are often cumbersome and inefficient for large-scale chloroplast genome analysis. As a result, conventional methods were not suitable for the 89 samples analyzed in this study. To overcome this limitation, we developed a pipeline for batch assembly, annotation, and downstream analysis (**Figure 1**). This pipeline consists of a series of scripts implemented in a Python v3.10.17 environment, integrates modules such as Biopython v1.85 and Perl v5.32.1, and incorporates third-party tools including Trimmomatic, BLASTn, MISA, Tandem Repeats Finder, and vmatch. IRscope and TBtools were additionally used for visualization of specific outputs. The pipeline includes two major components, assembly/annotation and data analysis, with the latter comprising genome structure analysis, nucleotide diversity analysis, and phylogenetic analysis. Detailed procedures are described in the Materials and Methods section.

Genome structure analysis involved identification of IR boundaries and adjacent genes, repetitive sequence analysis, and calculation of basic genomic features. Previous studies have commonly used GeSeq for IR boundary identification, but this visualization-based tool is not well suited for batch analysis. Therefore, our pipeline uses BLASTn-based self-to-self genome alignment to extract IR information in batch mode. Comparison of the IR boundaries identified by our script with those annotated by GeSeq showed consistent results, supporting the accuracy of the method. In addition, using codon table information provided by Biopython, we implemented a script for batch RSCU calculation as an alternative to CodonW. Because repetitive sequence analysis is often carried out separately with multiple web-based tools, large-scale processing can be cumbersome. By integrating several command-line tools into a single script, our pipeline enables efficient repetitive-sequence analysis across many genomes and markedly reduces processing time. Visualization was performed separately, with IRscope used for IR boundary display and TBtools used for RSCU heatmap generation.

For nucleotide diversity analysis, the pipeline adopted a region-based strategy rather than the sliding-window approach implemented in DnaSP (Rozas et al., 2017). Specifically, each chloroplast genome was first partitioned into individual CDSs and intergenic spacer (IGS) regions, and nucleotide diversity was then calculated separately for each region type. For coding regions, CDSs corresponding to the same gene were extracted from all samples, aligned using MAFFT, and Pi values were calculated for each gene across the entire dataset. Intergenic regions were more complex to analyze because structural variations, such as inversions, duplications, and deletions, could affect the consistency of region boundaries among genomes. To address this issue, gene names were first standardized to ensure that each intergenic region could be uniquely and consistently identified across all samples. Specifically, (1) standard gene names were retained according to the annotation files; (2) for genes represented by multiple fragmented copies (e.g., *rps12*), the fragments were named sequentially according to their genomic order (e.g., “*rps12*_part1” and “*rps12*_part2”); (3) if the same fragment name occurred more than once with different start positions, it was treated as a duplicated copy and renamed accordingly (e.g., two copies of “*rps12*_part2” were designated “*rps12*_part2” and “*rps12*_part2_copy1”); and (4) if two adjacent genes overlapped, or if the spacer between them was shorter than 10 bp, they were treated as a merged gene unit (e.g., *matK* located within *trnH*-UGU was designated “*trnH*-UGU-merge-*matK*”). After all intergenic regions had been extracted, a reference genome, typically the first genome in the dataset, was selected. Intergenic regions present in both the reference genome and the other samples were grouped for alignment and subsequent Pi calculation. Regions detected in non-reference genomes but absent from the reference were output separately as a file containing structural-variation information. Nucleotide diversity for each aligned intergenic region was then calculated following the same procedure used for the CDS regions.

To facilitate reproducibility and practical implementation, we further specified the input and output file types, key execution settings, and output formats of the major modules in the pipeline. The first three preprocessing steps—file renaming, data merging, and quality control—take raw read files from an input directory and write the processed results to corresponding output directories. These modules can automatically detect files in fastq.gz, fastq, fq.gz, or fq format within the input directory and process them in batch mode. For data quality control, Trimmomatic uses six threads by default, although users may specify a larger number of threads to accelerate execution. To enable large-scale chloroplast genome assembly using GetOrganelle, we developed a Python script that identifies “.paired.fastq.gz” files in the quality-control output directory, performs multi-sample assembly, and consolidates the assembly results of different samples into a single directory. In addition to the directory containing the genomes, all three steps require as input a GenBank file of the chloroplast genome from a reference species. Both strand orientation determination and start-position correction are implemented using BLASTn. To reduce processing time, these two scripts use 64 threads by default, thereby enabling rapid execution. Once genome annotation has been completed, downstream analyses can be performed. The input and output requirements for these analyses are relatively straightforward: all downstream modules take the annotated GenBank files as input and support batch processing using directories as input. With the exception of visualization of IR boundaries and codon usage bias, which must be performed locally using third-party tools to generate figure files, all other analyses produce text-based output files.

### 3.2 Structural analysis of chloroplast genomes

**Figure 2:**
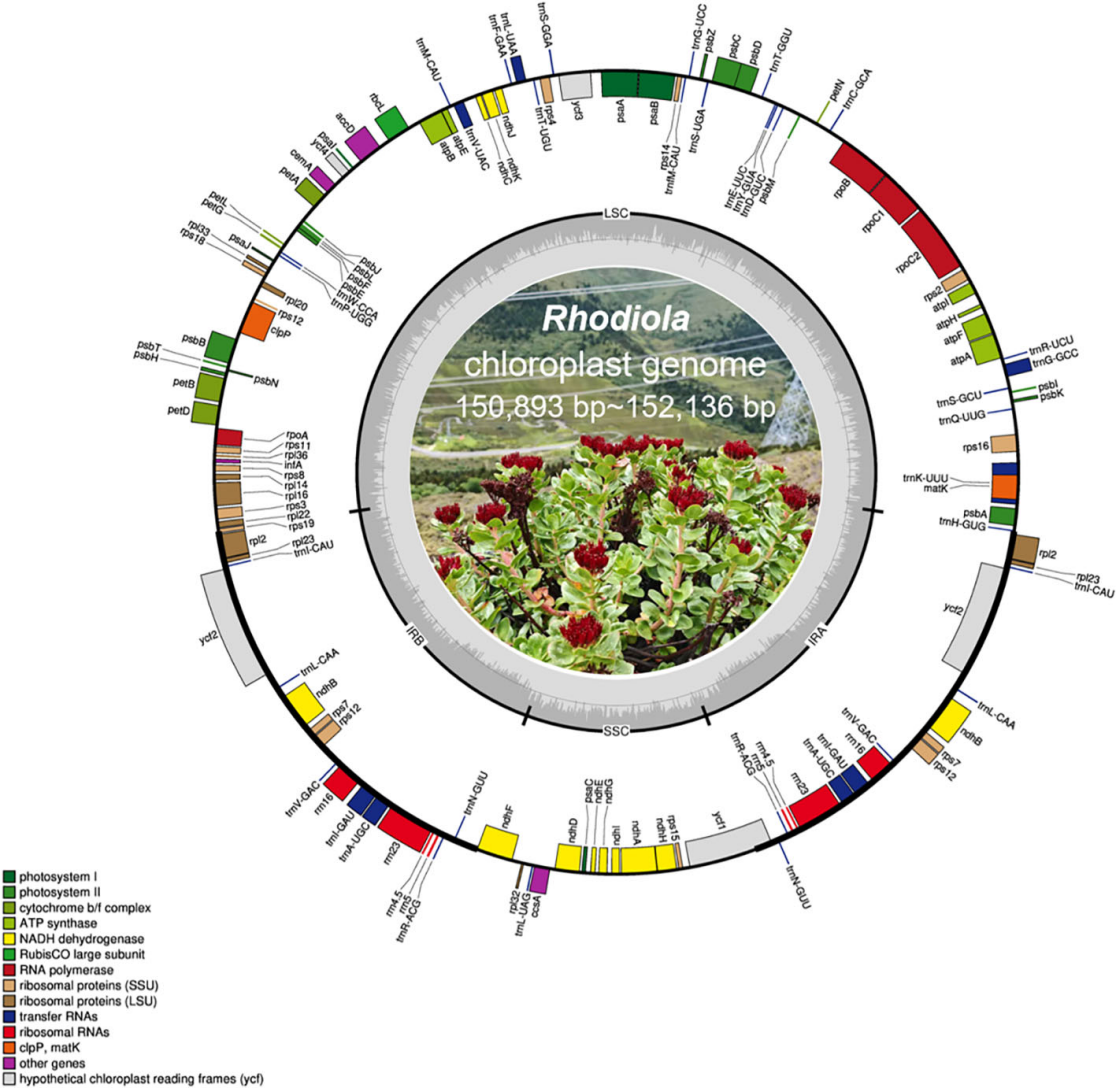
Gene map of 89 *Rhodiola* samples. Genes drawn outside the circle are transcribed clockwise, while those inside are transcribed counterclockwise. Genes are color-coded according to functional groups. The inner circle indicates the boundaries of the large single-copy (LSC), small single-copy (SSC), and two inverted repeat (IRa and IRb) regions, with GC and AT content shown in dark gray and light gray, respectively.

The chloroplast genomes of *Rhodiola* exhibit the typical quadripartite structure, comprising a large single-copy (LSC) region, a small single-copy (SSC) region, and two inverted repeat (IR) regions (**Figure 2**). Comparative analysis of 89 samples revealed that the overall chloroplast genome architecture is highly conserved across the genus, with only slight variation in genome size (**Supplementary Table 2**). The total genome length ranged from 150,893 bp to 152,136 bp. Specifically, the LSC region varied from 82,071 bp to 83,330 bp, the SSC region from 16,989 bp to 17,178 bp, and the IR regions were comparatively stable, ranging from 25,690 bp to 25,941 bp, with an average length of approximately 25,873 bp. The limited extent of size variation across all regions further highlights the structural conservation of the chloroplast genomes. Analysis of GC content showed that the overall GC content of *Rhodiola* chloroplast genomes ranged from 37.65% to 37.79%, indicating extremely low interspecific and intraspecific variation. Owing to the presence of GC-rich rRNA genes in the IR regions, the GC content of the IR regions (approximately 42.9%) was markedly higher than that of the LSC (approximately 35.7%) and SSC (approximately 31.6%) regions. In terms of gene composition, all sampled chloroplast genomes contained the same gene categories and numbers. Each genome was annotated with a total of 130 genes (**Supplementary Table 3**), including 85 protein-coding genes (PCGs), 37 transfer RNA (tRNA) genes, and 8 ribosomal RNA (rRNA) genes (*rrn23S, rrn16S, rrn5S*, and *rrn4.5S*). Due to the presence of the inverted repeat regions, several genes occurred in duplicate, including 6 PCGs (*ycf2, rpl2, rpl23, rps12, rps7*, and *ndhB*), 4 rRNA genes (*rrn16S, rrn23S, rrn4.5S*, and *rrn5S*), and 7 tRNA genes (*trnA-*UGC*, trnI-*CAU*, trnI-*GAU*, trnL-*CAA*, trnN-*GUU*, trnV-*GAC, and *trnR-*ACG). Across all samples, the proportion of protein-coding regions relative to the total genome length ranged from 48.19% to 51.91%, with most samples exceeding 51.5%. Together, these results indicate that the chloroplast genomes of *Rhodiola* are highly conserved in genome size, GC content, gene content, and overall structure, with only limited variation both among and within species, consistent with the general characteristics of angiosperm chloroplast genomes.

**Figure 3:**
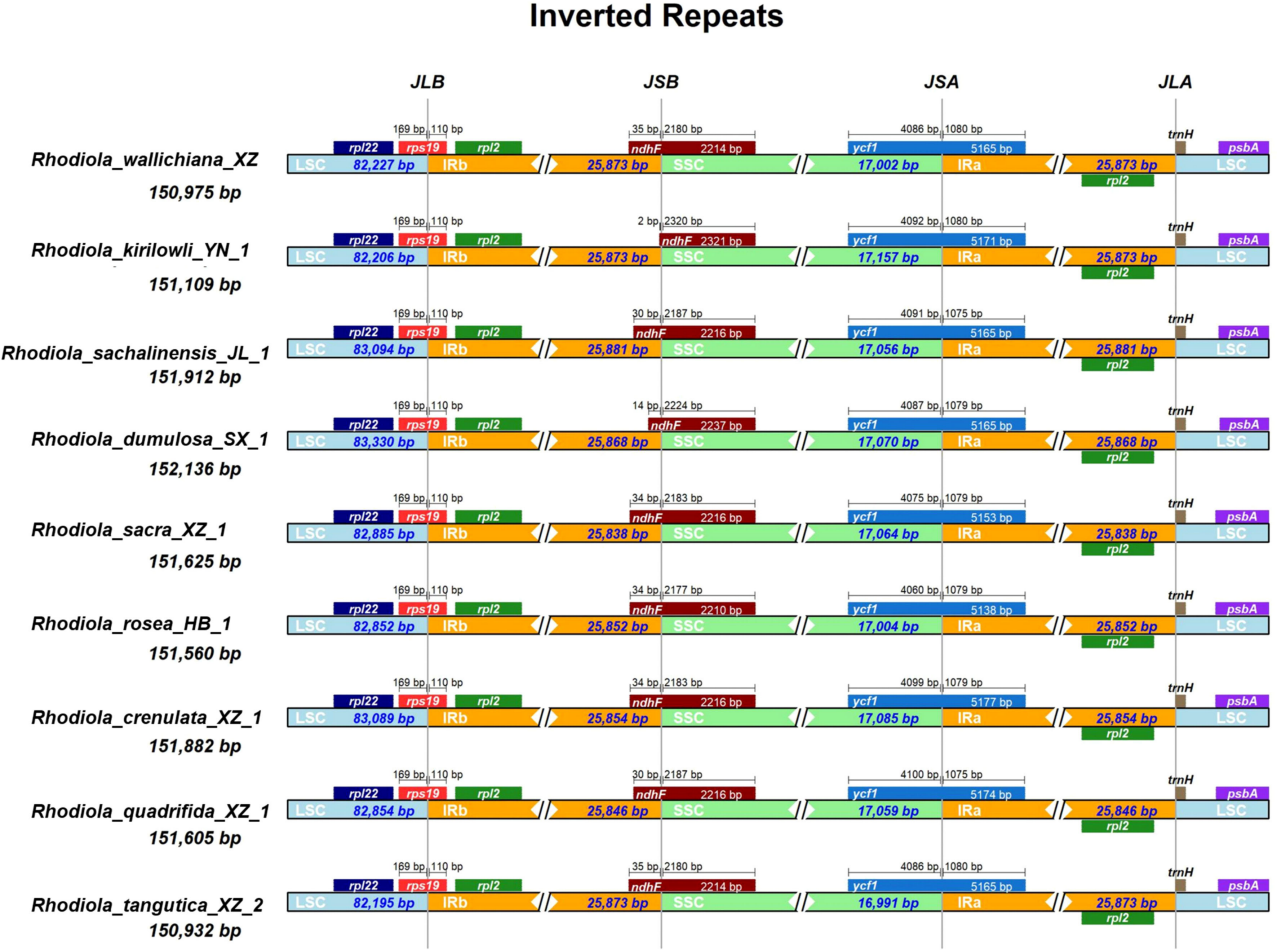
Comparison of IR boundary regions among nine representative medicinal *Rhodiola* species included in the Chinese Pharmacopoeia and regional medicinal standards. The diagram illustrates the junctions between the IR regions (IRa and IRb) and the single-copy regions (LSC and SSC). Genes located at or near the boundary sites are shown in colored boxes, with the distances (in base pairs) from the boundary indicated above the gene boxes. The direction of transcription is denoted by arrows. The expanded regions highlight the specific genes spanning the LSC/IRb, IRb/SSC, SSC/IRa, and IRa/LSC junctions. The scale is proportional to the length of the corresponding genomic regions.

To investigate structural variations in the chloroplast genomes of *Rhodiola*, we conducted a comparative analysis of the IR boundary regions and the distribution patterns of adjacent genes. For representative visualization, nine medicinally important *Rhodiola* species recorded in the Chinese Pharmacopoeia and regional medicinal standards were selected for IR boundary comparison using IRscope (**Figure 3**). In addition, the boundary gene positions and adjacent gene arrangements of all sampled chloroplast genomes were systematically examined, and the complete results are provided in **Supplementary Tables 4** and **5**. The comparative analysis showed that the overall genome structure remained highly conserved across all samples, with no detectable gene rearrangements. Boundary analysis between the IR and single-copy regions indicated that the LSC/IRb (JLB) and IRa/LSC (JLA) boundaries were relatively stable across all samples, showing no significant expansion or contraction. However, certain length variations were observed at the IRb/SSC (JSB) and SSC/IRa (JSA) boundaries. These variations were primarily attributed to length polymorphisms in the 3’-end repeat sequences of the *ndhF* and *ycf1* genes, resulting in slight shifts in stop codon positions.

### 3.3 Repetitive sequence characterization and codon usage bias analysis

Analysis of repetitive sequences across 89 *Rhodiola* chloroplast genomes revealed generally similar compositional patterns among samples, although some variation in repeat type and copy number was observed (**Supplementary Table 6**). The total number of simple sequence repeats (SSRs) per sample ranged from 43 to 59, with mononucleotide repeats being predominant (33–46 repeats). Dinucleotide repeats represented the second most abundant category (4–9 repeats), while tetranucleotide repeats remained relatively stable (3–5 repeats in most samples), with only *R. bupleuroides*_XZ_1 exhibiting a slightly higher count (6 repeats). Trinucleotide, pentanucleotide, and hexanucleotide repeats were considerably rarer, detected in only 18, 17, and 7 samples, respectively, typically with only one or two copies per sample. Hexanucleotide repeats were present exclusively in seven samples and showed inconsistent distribution among different populations of the same species, suggesting intraspecific polymorphism rather than species-specificity. Pentanucleotide repeats were similarly restricted to a limited number of samples, with *R. bupleuroides*_XZ_1 containing the highest count (two repeats). Tandem repeat analysis (TRF) identified 14 to 29 repeats per sample, with *R. rosea*_HB_1 displaying the highest number (29 repeats) and *R. dumulosa*_SX_1 exhibiting the lowest (14 repeats). Dispersed repeat analysis revealed considerable variation in palindromic repeats (5–53 repeats), with *R. rosea*_HB_1 showing a markedly high count of 53 repeats, substantially exceeding those of other samples. Forward repeats ranged from 8 to 25 per sample and were relatively stable, with *R. crenulata* (XZ_1, XZ_2) reaching the highest count (25 repeats) (**Supplementary Table 7**).

**Figure 4:**
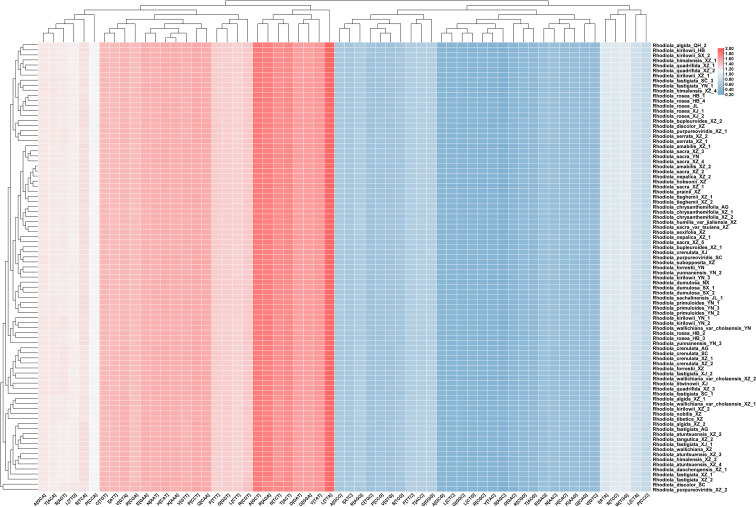
Heatmap analysis for RSCU values of all PCGs of 89 chloroplast genomes. Red and blue indicate higher and lower RSCU values, respectively.

The codon usage patterns of PCGs in the chloroplast genomes of *Rhodiola* were systematically investigated by calculating relative synonymous codon usage (RSCU) values (**Figure 4**). The total number of codons across individual chloroplast genomes ranged from 23,421 (*R. purpureoviridis*_XZ_2) to 26,122 (*R. quadrifida*_XZ_3). A pronounced codon usage bias was observed throughout the genus, with 30 codons exhibiting RSCU values greater than 1. Notably, 29 of these preferentially used codons terminated with A or U, demonstrating a clear bias toward A/U-ending codons. Conversely, codons ending with G or C predominantly displayed lower usage frequencies (RSCU < 1). This pattern was consistently exemplified across multiple amino acids. For alanine (Ala), among its four synonymous codons, GCT showed consistently high RSCU values (RSCU > 1.79), whereas GCG exhibited markedly lower usage (RSCU < 0.40). For leucine (Leu), which is encoded by six codons, TTA displayed the highest RSCU value among all codons examined, further underscoring the A/U preference. These codon usage patterns were highly consistent across different *Rhodiola* species, suggesting that the chloroplast genomes within this genus are subject to similar evolutionary constraints, likely related to translational efficiency optimization and regulatory mechanisms governing chloroplast gene expression. Only minor variations in RSCU values were observed among individual species, potentially reflecting incipient genetic differentiation between species or geographic populations. This overall conservation in codon usage bias implies strong purifying selection acting on translational dynamics within the chloroplast.

### 3.4 Genetic diversity in genes and intergenic spacers

**Figure 5:**
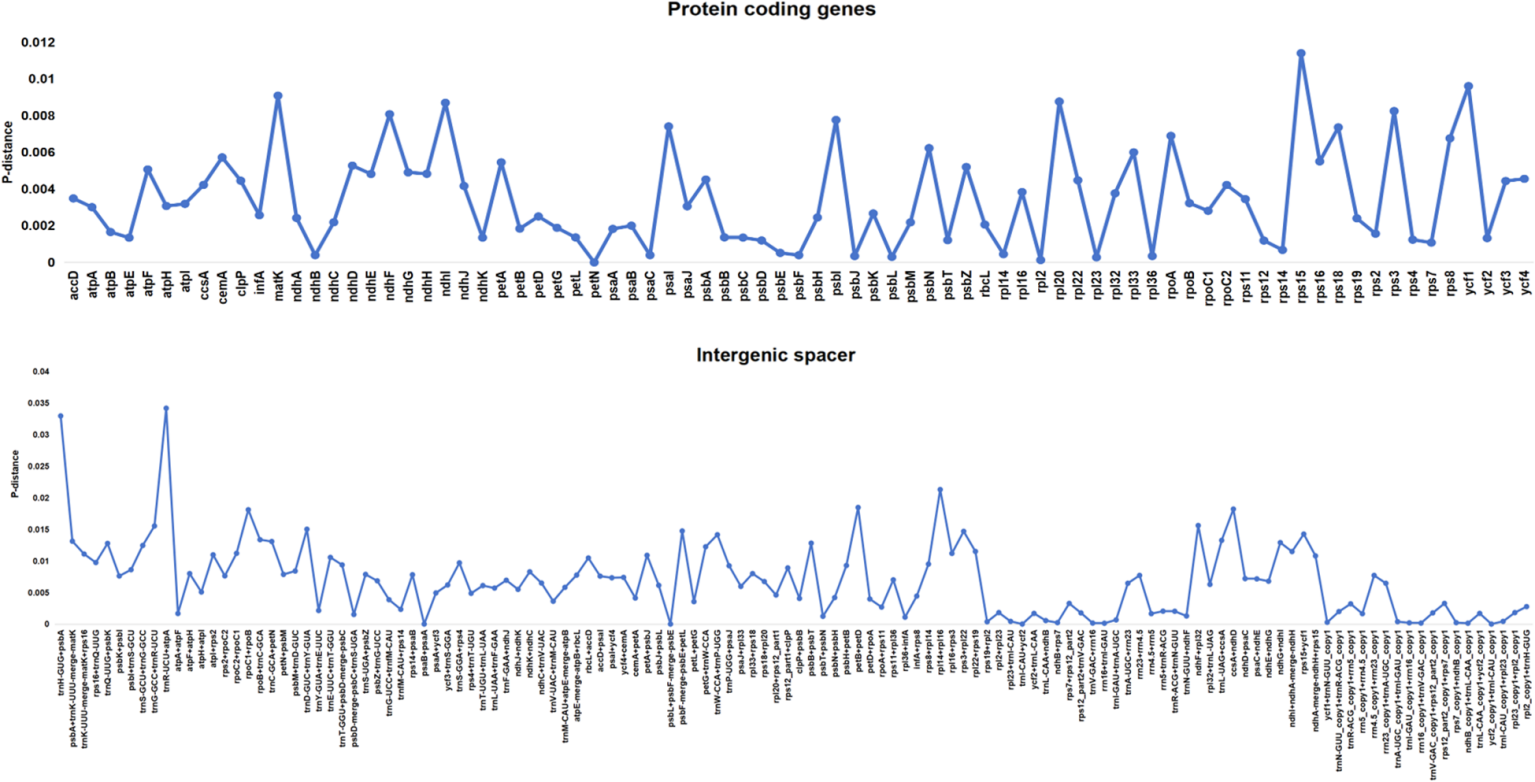
Comparison of nucleotide variability (Pi) values for PCGs and intergenic regions.

Based on the Pi analysis of 89 *Rhodiola* chloroplast genomes, we assessed the degree of sequence variation in both intergenic spacers (IGSs) and PCGs (**Figure 5**; **Supplementary Table 8**). The results revealed substantial variation in Pi values across different regions. For PCGs, Pi values ranged from 0 to 0.0114. The gene exhibiting the highest level of variation was *rps15*, followed by *ycf1*, *matK*, *rpl20*, *ndhI*, and *rps3*. These genes represent promising candidate high-variation markers for future phylogenetic reconstruction and DNA barcoding studies within the genus *Rhodiola*. In contrast, most core genes involved in photosynthesis (e.g., *psaA*, *psbA*, *rbcL*) exhibited Pi values generally below 0.005, indicating strong evolutionary conservation. Genes located within the inverted repeat (IR) regions (e.g., *rpl2*, *rpl23*, *ycf2*, *ndhB*) showed Pi values approaching zero, further confirming the stability of the IR regions during genome evolution. For intergenic regions, Pi values ranged from 0 to 0.034. The region with the highest nucleotide diversity was *trnR-*UCU*-atpA*, followed by *trnH-*GUG*-psbA* and *rpl14-rpl16*. Additionally, regions such as *rpoC1-rpoB*, *ccsA-ndhD* and *petB-petD* also displayed relatively high levels of polymorphism. Notably, during the alignment process, a significant length expansion was observed in the intergenic spacer between *ndhA* and *ndhH* in the *Rhodiola discolor*_XZ sample. This region was approximately 108 bp in length, whereas the corresponding interval in the majority of other samples was only about 2 bp long. This structural variation contributed to the moderate Pi value (0.01147) calculated for this region and suggests the presence of a potential species-specific insertion event within the genus *Rhodiola*. In contrast, IGSs located in the IR regions (e.g., *trnI-*CAU*-ycf2*, *rrn16-trnI-*GAU) exhibited Pi values approaching zero, reflecting their high degree of conservation. Additionally, several lineage-specific polymorphic sites were identified as candidate chloroplast genome markers of maternal lineage divergence associated with sexual-system evolution. Polymorphic loci in *rps15* (site 87), *ndhI* (sites 21, 162, and 283), *rps3* (sites 84, 160, and 207), and *rpl20* (sites 33 and 165) separated the samples into two distinct lineages corresponding to hermaphroditic and dioecious groups. Single nucleotide polymorphism (SNP) analysis was performed on chloroplast PCGs across *Rhodiola* accessions based on the assembled chloroplast genome data (**Supplementary Table 9**). The results revealed substantial variation in SNP distribution among genes, with *ycf1* exhibiting by far the highest SNP count (460 SNPs), underscoring its status as the most variable region within the *Rhodiola* chloroplast genome. Several other genes also displayed elevated genetic diversity, including *rpoC2* (203 SNPs), *ndhF* (216 SNPs), *rpoB* (115 SNPs), and *matK* (122 SNPs). In marked contrast, the *petN* gene, despite its coding sequence length of 90 bp, remained completely conserved across all accessions with no detectable SNP sites. This extreme conservation suggests that *petN* likely performs an essential housekeeping function and is subject to strong purifying selection. Similarly, several photosynthesis-related genes exhibited high sequence conservation, including *psbF* (2 SNPs), *psbL* (4 SNPs), *psbJ* (4 SNPs), and *atpH* (3 SNPs). The proteins encoded by these genes participate in core photosystem II assembly and functional maintenance, and their pronounced sequence conservation is consistent with their functional importance in photosynthetic machinery.

### 3.5 Phylogenetic analysis and sex differentiation

**Figure 6:**
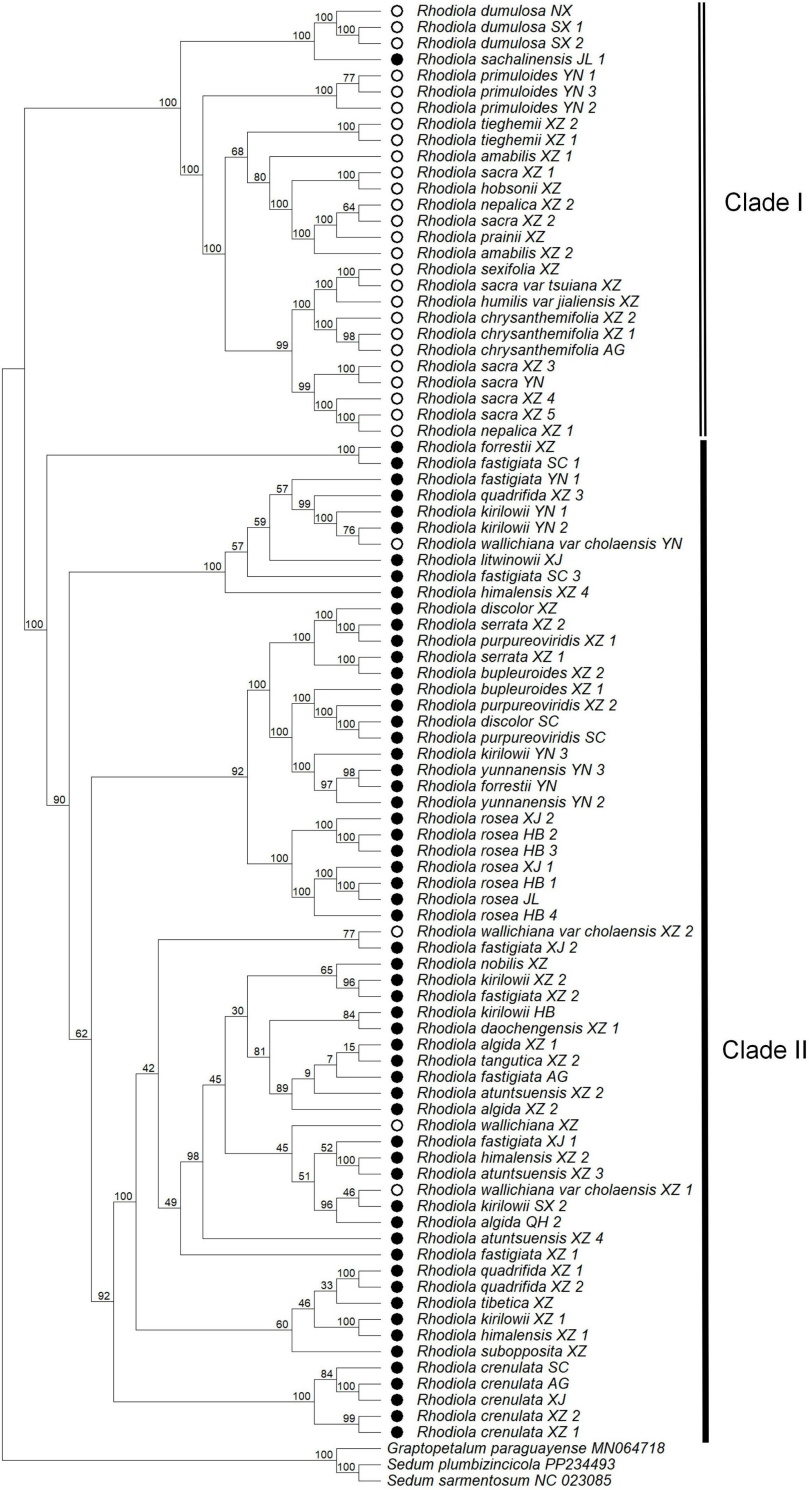
Phylogenetic tree based on concatenated chloroplast CDS sequences from 89 samples. Open circles represent hermaphroditic individuals, while solid black circles denote dioecious individuals.

A phylogenetic tree was reconstructed using the ML method based on chloroplast genome data from 89 samples, with three Crassulaceae species—*Graptopetalum paraguayense*, *Sedum plumbizincicola*, and *Sedum sarmentosum*—used as outgroups (**Figure 6**). Most nodes received strong support, with bootstrap values exceeding 95% for the majority of branches. The resulting phylogeny clearly resolved two major clades with 99% bootstrap support, broadly corresponding to hermaphroditic and dioecious lineages. The hermaphroditic clade occupied the more basal position in the rooted tree, a pattern consistent with the hypothesis that dioecy in *Rhodiola* was derived from hermaphroditic ancestors. The early divergence of these two major lineages was strongly supported, suggesting a close association between sexual-system differentiation and early lineage divergence in *Rhodiola*. Regarding clade composition, Clade I consisted predominantly of hermaphroditic species, with the exception of *R. sachalinensis* from Jilin. This clade included multiple populations of *R. dumulosa*, *R. chrysanthemifolia*, and *R. himalensis*, among others. Clade II was composed almost exclusively of dioecious species, including *R. rosea*, *R. yunnanensis*, *R. crenulata*, and their allies, with the exception of *R. wallichiana* and its variety *R. wallichiana* var. *cholaensis*. The monophyly of core species groups was strongly supported by the chloroplast phylogeny. Seven samples of *R. rosea* from distinct geographical populations formed a well-supported monophyletic clade with 99% bootstrap support. Similarly, six populations of *R. crenulata* clustered together with 99% bootstrap support. Species such as *R. chrysanthemifolia* and *R. primuloides* also exhibited clear monophyly. These results indicate that the aforementioned species are well-delimited both morphologically and genetically, with relatively stable genetic structures. In contrast, multiple populations of *R. sacra*, *R. kirilowii*, *R. fastigiata*, and *R. wallichiana* var. *cholaensis* displayed polyphyly, with different geographical populations scattered across distinct phylogenetic branches. This pattern suggests that geographical isolation may have promoted independent differentiation of genetic lineages within these species, resulting in complex population genetic structures.

## 4 Discussion

In this study, we comprehensively analyzed the chloroplast genomes of 89 *Rhodiola* samples representing 35 species and revealed variation patterns at multiple levels, including sequence divergence, repeat composition, and phylogenetic structure. Overall, the chloroplast genomes of *Rhodiola* were relatively conserved, but several loci showed pronounced lineage-specific variation, indicating that chloroplast genomes still retain informative evolutionary signals despite their structural stability. More importantly, these signals were not randomly distributed; rather, they were associated with the major phylogenetic split within the genus and appeared to be broadly correlated with divergence in sexual systems. A notable result was that several genes exhibited relatively few SNPs but comparatively high Pi values, suggesting that their differentiation was not caused by the gradual accumulation of numerous low-frequency mutations, but instead by a limited number of diagnostic substitutions that became fixed or highly frequent in different lineages. Among these genes, *rps15* was particularly striking, showing the highest nucleotide diversity among all PCGs, with site 87 displaying a pattern highly consistent with the separation between dioecious and hermaphroditic taxa. Similar lineage-discriminating patterns were also detected in *ndhI* (sites 21, 162, and 283), *rps3* (sites 84, 160, and 207), and *rpl20* (sites 33 and 165). Taken together, these loci are more reasonably interpreted as chloroplast markers associated with maternal lineage divergence accompanying sexual-system evolution, rather than as direct determinants of sex differentiation (Thureborn et al., 2024). Future studies integrating nuclear genomic data with broader population sampling will be necessary to disentangle the relative contributions of sexual-system divergence, lineage history, and ecological adaptation to the formation of these hotspot loci.

The repeat sequence analysis provides another layer of evidence for the conservative yet informative nature of *Rhodiola* chloroplast genomes. Across the 89 samples, SSR composition was broadly similar, with mononucleotide repeats clearly dominating and dinucleotide repeats ranking second, whereas trinucleotide, pentanucleotide, and hexanucleotide repeats were much less frequent. In particular, trinucleotide SSRs were detected in only a small subset of samples, which is consistent with the general pattern reported for plant chloroplast genomes. In nuclear genomes, especially in coding or transcribed regions, trinucleotide repeats are often preferentially retained because variation in repeat number does not disrupt the reading frame; by contrast, changes in repeats with motif lengths not divisible by three are more likely to cause frameshift mutations and are therefore more strongly constrained by purifying selection (Li et al., 2004). However, chloroplast SSRs are typically enriched in noncoding regions and are overwhelmingly dominated by A/T-rich mononucleotide motifs (Duan et al., 2020). Therefore, the rarity of trinucleotide repeats in *Rhodiola* chloroplast genomes likely reflects both the highly conserved nature of chloroplast genomes and the stronger structural constraints acting on plastid coding sequences. Although these rare trinucleotide repeats may not carry obvious functional significance, they may still be useful as supplementary molecular markers for haplotype identification, intraspecific polymorphism detection, and phylogeographic analysis.

The phylogenetic tree reconstructed from chloroplast CDS data of 89 samples representing 35 *Rhodiola* species was largely consistent with the topology reported in previous studies based on 23 representative species (Zhao et al., 2021). The genus was resolved into two major clades: one predominantly comprising hermaphroditic species and the other consisting entirely of dioecious species. This result further demonstrates the effectiveness of chloroplast genome data in resolving phylogenetic relationships among recently radiated lineages and highlights a close association between sexual system differentiation and early lineage diversification in *Rhodiola*. Notably, several samples exhibited phylogenetic placements inconsistent with the predominant sexual system of their respective clades, including *R. sachalinensis*, *R. wallichiana*, and *R. wallichiana* var. *cholaensis*. These discordant placements suggest that these lineages may have undergone independent trajectories of sex differentiation or experienced complex reticulate evolutionary processes. In addition, repeated independent origins of dioecy within *Rhodiola* have been proposed, raising the possibility that *R. sachalinensis* represents an independent transition in sexual-system evolution (Zhao et al., 2021). In our phylogeny, *R. wallichiana* and its variety, *R. wallichiana* var. *cholaensis*, were placed within the dioecious clade in our phylogeny, despite being described in the *Flora of China* as predominantly hermaphroditic with occasional dioecious individuals. Recent work has provided insights into the evolutionary pathway underlying the transition from hermaphroditism to dioecy. Lesaffre et al. (2024) showed that under selective pressure from inbreeding depression, male-sterility mutations may initially spread in hermaphroditic populations, followed by the fixation of female-sterility mutations, ultimately resulting in stable dioecy. During this process, transitional states in which hermaphroditic and dioecious individuals coexist may persist. *R. wallichiana* and its variety may represent such transitional lineages, retaining both sexual systems while phylogenetically nested within the dioecious clade.

Evidence from nuclear genomic studies further supports the association between sexual system differentiation and genomic evolution in *Rhodiola*. Chromosome-level genomic comparisons between two species representing different sexual systems—hermaphroditic *R. chrysanthemifolia* and dioecious *R. kirilowii*—revealed substantial differences in genome size, transposable element composition, and chromosomal architecture (Zhang et al., 2024). These findings suggest that sexual system divergence is accompanied by structural genomic variation at the nuclear level. Our phylogenetic analysis provides complementary macroevolutionary evidence, indicating that sex differentiation has left detectable phylogenetic signatures throughout the evolutionary history of *Rhodiola*. Future studies integrating nuclear genomic data with broader population sampling, and considering evolutionary mechanisms such as chloroplast capture and hybridization–introgression, will be essential for fully elucidating the evolutionary dynamics of sex differentiation in this alpine genus.

## 5 Conclusion

In this study, we developed a batch-processing pipeline to comprehensively analyze the chloroplast genomes of 89 *Rhodiola* samples representing 35 species. Our results reveal that the chloroplast genomes are highly conserved in overall structure, gene content, and codon usage, yet exhibit moderate variation in specific genes and intergenic regions. Several highly variable regions were identified, including the coding genes *ycf1*, *matK*, *rpoC2*, and *rpoB*, as well as the intergenic regions *trnR*-UCU–*atpA*, *trnH*-GUG–*psbA*, and *rpl14–rpl16*, which may serve as informative molecular markers for species identification and phylogenetic reconstruction. Notably, a 108-bp expansion in the *ndhA*–*ndhH* intergenic region was detected exclusively in *R. discolor*, representing a rare structural variation within the genus. Phylogenetic analyses recovered two major clades broadly corresponding to hermaphroditic and dioecious lineages, although exceptions such as *R. sachalinensis*, *R. wallichiana*, and *R. wallichiana* var. *cholaensis* indicate a more complex evolutionary history involving parallel evolution. Furthermore, lineage-specific polymorphisms in the *rps15*, *ndhI*, *rps3*, and *rpl20* were identified as candidate chloroplast genome markers associated with maternal lineage divergence linked to sexual-system evolution, rather than as direct determinants of sex differentiation. These findings provide new insight into chloroplast genome evolution in *Rhodiola* and highlight candidate regions for future investigation of the evolutionary processes underlying reproductive-trait divergence. Integrative analyses combining nuclear and chloroplast genomic data will be essential to fully resolve the evolutionary dynamics of sexual systems in this genus.

## Data Availability

All custom scripts used in the batch-processing pipeline are available on GitHub (https://github.com/YiYiYiDa/Batch-Assembly-of-Chloroplast-Genomes).
