# Supplementary material for: Analysis of structural variation and sex differentiation associated phylogenetic signals in newly sequenced Rhodiola chloroplast genomes using a batch processing pipeline
Source: Front Plant Sci. 2026 May 11;17:1829588. doi: 10.3389/fpls.2026.1829588 (PMC13199353; doi:10.3389/fpls.2026.1829588)
Supplement: Supplementary file 1 [file Table1.doc]

***Supplementary Material***

**Supplementary Table 1.** The location information of 89 *Rhodiola* samples.

| Samples | Location |
| --- | --- |
| Rhodiola_algida_QH_2 | Banma County, Zhuluo Prefecture, Qinghai |
| Rhodiola_algida_XZ_1 | Jiegar, Leiniaoqi County, Qamdo City, Tibet |
| Rhodiola_algida_XZ_2 | Langkazi County, Shannan City, Tibet |
| Rhodiola_amabilis_XZ_1 | Chidou Town, Dingqing County, Qamdo City, Tibet |
| Rhodiola_amabilis_XZ_2 | Mozhugongkar County, Lhasa City, Tibet |
| Rhodiola_atuntsuensis_XZ_2 | Mugabu, Langkazi County, Shannan City, Tibet |
| Rhodiola_atuntsuensis_XZ_3 | Chapu, Gongbujiangda County, Nyingchi City, Tibet |
| Rhodiola_atuntsuensis_XZ_4 | National Highway 318, Linzhi Town, Bayi District, Linzhi City, Tibet |
| Rhodiola_bupleuroides_XZ_1 | Zangbaka, Mangkang County, Qamdo City, Tibet |
| Rhodiola_bupleuroides_XZ_2 | Lalong Village Bridge, G4218 Linla Highway, Mozhugongka County, Lhasa City, Tibet |
| Rhodiola_chrysanthemifolia_AG | Anguo medicinal materials market, Baoding, Hebei |
| Rhodiola_chrysanthemifolia_XZ_1 | Bayi District, Linzhi City, Tibet |
| Rhodiola_chrysanthemifolia_XZ_2 | National Highway 318, Lulang Town, Bayi District, Nyingchi City, Tibet |
| Rhodiola_crenulata_AG | Anguo medicinal materials market, Baoding, Hebei |
| Rhodiola_crenulata_SC | Three peaks in Changpinggou Scenic Area, Siguniangshan Town, Xiaojin County, Aba Prefecture, Sichuan |
| Rhodiola_crenulata_XJ | Xinjiang |
| Rhodiola_crenulata_XZ_1 | Gongbu Jiangda County, Linzhi City, Tibet |
| Rhodiola_crenulata_XZ_2 | Chapu, Gongbujiangda County, Nyingchi City, Tibet |
| Rhodiola_daochengensis_XZ_1 | Entrance of G6 Beijing-Tibet Expressway (Nakqu direction), Doulungdeqing District, Lhasa City, Tibet |
| Rhodiola_discolor_SC | Zhabu, Litang County, Garze Tibetan Autonomous Prefecture, Sichuan |
| Rhodiola_discolor_XZ | Bibu County, Nagqu City, Tibet |
| Rhodiola_dumulosa_NX | Sandaogou, Helan Mountain, Yinchuan, Ningxia |
| Rhodiola_dumulosa_SX_1 | Taibai Mountain, Shaanxi |
| Rhodiola_dumulosa_SX_2 | Taibai Mountain, Shaanxi |
| Rhodiola_fastigiata_AG | Anguo medicinal materials market, Baoding, Hebei |
| Rhodiola_fastigiata_SC_1 | Jianziwanshan Tunnel, Yajiang County, Garze Tibetan Autonomous Prefecture, Sichuan |
| Rhodiola_fastigiata_SC_3 | Litang County, Garze Tibetan Autonomous Prefecture, Sichuan |
| Rhodiola_fastigiata_XJ_1 | Xinjiang |
| Rhodiola_fastigiata_XJ_2 | Xinjiang |
| Rhodiola_fastigiata_XZ_1 | Tibet |
| Rhodiola_fastigiata_XZ_2 | Zuogong County, Qamdo City, Tibet |
| Rhodiola_fastigiata_YN_1 | National Highway 214, Shengping Town, Deqin County, Diqing Tibetan Autonomous Prefecture, Yunnan |
| Rhodiola_forrestii_XZ | Sejila Mountain, Bayi District, Nyingchi City, Tibet |
| Rhodiola_forrestii_YN | Lijiang City, Yunnan |
| Rhodiola_himalensis_XZ_1 | Bazongwa, Chaya County, Qamdo City, Tibet |
| Rhodiola_himalensis_XZ_2 | Chaga Bridge (Hala Bridge), Suo County, Nagqu City, Tibet |
| Rhodiola_himalensis_XZ_4 | Bomi County, Linzhi City, Tibet |
| Rhodiola_hobsonii_XZ | Bado Middle Bridge, Dingqing County, Qamdo City, Tibet |
| Rhodiola_humilis_var_jialiensis_XZ | Shangshang Village, Dingqing County, Qamdo City, Tibet |
| Rhodiola_kirilowii_HB | Weichang, Hebei |
| Rhodiola_kirilowii_SX_2 | Taibai Mountain, Shaanxi |
| Rhodiola_kirilowii_XZ_1 | Chamang Highway, Chaya County, Qamdo City, Tibet |
| Rhodiola_kirilowii_XZ_2 | Bado Middle Bridge, Dingqing County, Qamdo City, Tibet |
| Rhodiola_kirilowii_YN_1 | Shangri-La City, Diqing Prefecture, Yunnan |
| Rhodiola_kirilowii_YN_2 | Deqin County, Diqing Tibetan Autonomous Prefecture, Yunnan |
| Rhodiola_kirilowii_YN_3 | National Highway 214, Shengping Town, Deqin County, Diqing Tibetan Autonomous Prefecture, Yunnan |
| Rhodiola_litwinowii_XJ | Taxkorgan Tajik Autonomous County, Kashgar District, Xinjiang |
| Rhodiola_nepalica_XZ_1 | Xingna, Qusong County, Shannan City, Tibet |
| Rhodiola_nepalica_XZ_2 | Bori, Qusong County, Shannan City, Tibet |
| Rhodiola_nobilis_XZ | National Highway 318, Lulang Town, Bayi District, Nyingchi City, Tibet |
| Rhodiola_prainii_XZ | Entrance of G6 Beijing-Tibet Expressway (Nakqu direction), Doulungdeqing District, Lhasa City, Tibet |
| Rhodiola_primuloides_YN_1 | Jianchuan County, Dali Prefecture, Yunnan |
| Rhodiola_primuloides_YN_2 | Eryuan County, Dali Bai Autonomous Prefecture, Yunnan |
| Rhodiola_primuloides_YN_3 | Eryuan County, Dali Bai Autonomous Prefecture, Yunnan |
| Rhodiola_purpureoviridis_SC | Litang County, Garze Tibetan Autonomous Prefecture, Sichuan |
| Rhodiola_purpureoviridis_XZ_1 | Jiongbuqiong, Qusong County, Shannan City, Tibet |
| Rhodiola_purpureoviridis_XZ_2 | Chaya Highway, Xiangdui Town, Chaya County, Qamdo City, Tibet |
| Rhodiola_quadrifida_XZ_1 | Zangbaka, Mangkang County, Qamdo City, Tibet |
| Rhodiola_quadrifida_XZ_2 | Zangbaka, Mangkang County, Qamdo City, Tibet |
| Rhodiola_quadrifida_XZ_3 | Qamdo District, Tibet |
| Rhodiola_rosea_HB_1 | Dadanzi Township, Fengning County, Hebei |
| Rhodiola_rosea_HB_2 | Dadanzi Township, Fengning County, Hebei |
| Rhodiola_rosea_HB_3 | Dadanzi Township, Fengning County, Hebei |
| Rhodiola_rosea_HB_4 | Weichang, Hebei |
| Rhodiola_rosea_JL | Laobai Mountain, Dunhua City, Yanbian Korean Autonomous Prefecture, Jilin |
| Rhodiola_rosea_XJ_1 | Xinjiang |
| Rhodiola_rosea_XJ_2 | Taxkorgan Tajik Autonomous County, Kashgar District, Xinjiang |
| Rhodiola_sachalinensis_JL_1 | Tonghua, Jilin |
| Rhodiola_sacra_var_tsuiana_XZ | Chamang Highway, Chaya County, Qamdo City, Tibet |
| Rhodiola_sacra_XZ_1 | Reshapu, Suo County, Nagqu City, Tibet |
| Rhodiola_sacra_XZ_2 | Zhenri, Gonggar County, Shannan City, Tibet |
| Rhodiola_sacra_XZ_3 | Entrance of G6 Beijing-Tibet Expressway (Nakqu direction), Doulungdeqing District, Lhasa City, Tibet |
| Rhodiola_sacra_XZ_4 | National Highway 349, Gonggar County, Shannan City, Tibet |
| Rhodiola_sacra_XZ_5 | Mozhugongka County, Lhasa City, Tibet |
| Rhodiola_sacra_YN | Yunnan |
| Rhodiola_serrata_XZ_1 | Xingna, Qusong County, Shannan City, Tibet |
| Rhodiola_serrata_XZ_2 | Xingna, Xiajiang Township, Qusong County, Shannan City, Tibet |
| Rhodiola_sexifolia_XZ | Zhongji, Chaya County, Qamdo City, Tibet |
| Rhodiola_subopposita_XZ | Chapu, Gongbujiangda County, Nyingchi City, Tibet |
| Rhodiola_tangutica_XZ_2 | Entrance of G6 Beijing-Tibet Expressway (Nakqu direction), Doulungdeqing District, Lhasa City, Tibet |
| Rhodiola_tibetica_XZ | National Highway 317, Dingqing County, Qamdo City, Tibet, goes directly to Dhaka |
| Rhodiola_tieghemii_XZ_1 | Bayi District, Linzhi City, Tibet |
| Rhodiola_tieghemii_XZ_2 | Zhangba Village, Bayi District, Nyingchi City, Tibet |
| Rhodiola_wallichiana_var_cholaensis_XZ_1 | National Highway 318, Linzhi Town, Bayi District, Linzhi City, Tibet |
| Rhodiola_wallichiana_var_cholaensis_XZ_2 | Chagang'er, Bayi District, Nyingchi City, Tibet |
| Rhodiola_wallichiana_var_cholaensis_YN | Deqin County, Diqing Prefecture, Yunnan |
| Rhodiola_wallichiana_XZ | Bayi District, Linzhi City, Tibet |
| Rhodiola_yunnanensis_YN_2 | Liangwang Mountain, Chenggong District, Kunming City, Yunnan |
| Rhodiola_yunnanensis_YN_3 | Kunming, Yunnan |

**Supplementary Table 2**. Assembly and annotation statistics of the *Rhodiola* chloroplast genomes.

| Genome_name | size(bp) | LSC(bp) | IRs(bp) | SSC(bp) | GC_content | Total_genes | rRNA_genes | tRNA_genes | Protein_coding_genes | Protein_coding_region(%) |
| --- | --- | --- | --- | --- | --- | --- | --- | --- | --- | --- |
| Rhodiola_algida_QH_2 | 151019 | 82245 | 25873 | 17016 | 37.76% | 130 | 8 | 37 | 85 | 51.89% |
| Rhodiola_algida_XZ_1 | 151079 | 82339 | 25873 | 16994 | 37.76% | 130 | 8 | 37 | 85 | 51.83% |
| Rhodiola_algida_XZ_2 | 150961 | 82221 | 25873 | 16994 | 37.77% | 130 | 8 | 37 | 85 | 51.87% |
| Rhodiola_amabilis_XZ_1 | 151630 | 82804 | 25872 | 17082 | 37.70% | 130 | 8 | 37 | 85 | 51.64% |
| Rhodiola_amabilis_XZ_2 | 151648 | 82878 | 25854 | 17062 | 37.72% | 130 | 8 | 37 | 85 | 51.63% |
| Rhodiola_atuntsuensis_XZ_2 | 150976 | 82235 | 25873 | 16995 | 37.76% | 130 | 8 | 37 | 85 | 51.87% |
| Rhodiola_atuntsuensis_XZ_3 | 151014 | 82268 | 25873 | 17000 | 37.75% | 130 | 8 | 37 | 85 | 51.85% |
| Rhodiola_atuntsuensis_XZ_4 | 150961 | 82204 | 25873 | 17011 | 37.78% | 130 | 8 | 37 | 85 | 51.87% |
| Rhodiola_bupleuroides_XZ_1 | 151827 | 83026 | 25870 | 17061 | 37.71% | 130 | 8 | 37 | 85 | 51.59% |
| Rhodiola_bupleuroides_XZ_2 | 151672 | 82846 | 25893 | 17040 | 37.70% | 130 | 8 | 37 | 85 | 51.66% |
| Rhodiola_chrysanthemifolia_AG | 151433 | 82637 | 25863 | 17070 | 37.74% | 130 | 8 | 37 | 85 | 51.71% |
| Rhodiola_chrysanthemifolia_XZ_1 | 151433 | 82637 | 25863 | 17070 | 37.74% | 130 | 8 | 37 | 85 | 51.71% |
| Rhodiola_chrysanthemifolia_XZ_2 | 151442 | 82646 | 25863 | 17070 | 37.74% | 130 | 8 | 37 | 85 | 51.71% |
| Rhodiola_crenulata_AG | 151907 | 83093 | 25883 | 17048 | 37.69% | 130 | 8 | 37 | 85 | 51.57% |
| Rhodiola_crenulata_SC | 151846 | 83043 | 25883 | 17037 | 37.70% | 130 | 8 | 37 | 85 | 51.59% |
| Rhodiola_crenulata_XJ | 151890 | 83076 | 25883 | 17048 | 37.69% | 130 | 8 | 37 | 85 | 51.57% |
| Rhodiola_crenulata_XZ_1 | 151912 | 83094 | 25884 | 17050 | 37.69% | 130 | 8 | 37 | 85 | 51.57% |
| Rhodiola_crenulata_XZ_2 | 151905 | 83088 | 25884 | 17049 | 37.69% | 130 | 8 | 37 | 85 | 51.57% |
| Rhodiola_daochengensis_XZ_1 | 150893 | 82234 | 25835 | 16989 | 37.75% | 130 | 8 | 37 | 85 | 51.90% |
| Rhodiola_discolor_SC | 151989 | 83053 | 25879 | 17178 | 37.71% | 130 | 8 | 37 | 85 | 50.90% |
| Rhodiola_discolor_XZ | 151798 | 83009 | 25824 | 17141 | 37.67% | 130 | 8 | 37 | 85 | 51.61% |
| Rhodiola_dumulosa_NX | 151541 | 82818 | 25843 | 17037 | 37.78% | 130 | 8 | 37 | 85 | 51.65% |
| Rhodiola_dumulosa_SX_1 | 151560 | 82852 | 25852 | 17004 | 37.75% | 130 | 8 | 37 | 85 | 51.64% |
| Rhodiola_dumulosa_SX_2 | 151575 | 82861 | 25852 | 17010 | 37.75% | 130 | 8 | 37 | 85 | 51.64% |
| Rhodiola_fastigiata_AG | 151061 | 82207 | 25873 | 17108 | 37.76% | 130 | 8 | 37 | 85 | 51.84% |
| Rhodiola_fastigiata_SC_1 | 151861 | 83053 | 25886 | 17037 | 37.70% | 130 | 8 | 37 | 85 | 51.63% |
| Rhodiola_fastigiata_SC_3 | 151814 | 83062 | 25850 | 17052 | 37.69% | 130 | 8 | 37 | 85 | 51.62% |
| Rhodiola_fastigiata_XJ_1 | 150972 | 82217 | 25873 | 17009 | 37.76% | 130 | 8 | 37 | 85 | 51.87% |
| Rhodiola_fastigiata_XJ_2 | 151067 | 82210 | 25874 | 17109 | 37.75% | 130 | 8 | 37 | 85 | 51.83% |
| Rhodiola_fastigiata_XZ_1 | 151010 | 82250 | 25874 | 17012 | 37.78% | 130 | 8 | 37 | 85 | 51.87% |
| Rhodiola_fastigiata_XZ_2 | 150915 | 82071 | 25873 | 17098 | 37.77% | 130 | 8 | 37 | 85 | 51.25% |
| Rhodiola_fastigiata_YN_1 | 151868 | 83081 | 25846 | 17095 | 37.70% | 130 | 8 | 37 | 85 | 51.59% |
| Rhodiola_forrestii_XZ | 151784 | 82997 | 25885 | 17017 | 37.71% | 130 | 8 | 37 | 85 | 51.61% |
| Rhodiola_forrestii_YN | 151681 | 82945 | 25855 | 17026 | 37.76% | 130 | 8 | 37 | 85 | 51.67% |
| Rhodiola_himalensis_XZ_1 | 151004 | 82209 | 25885 | 17025 | 37.77% | 130 | 8 | 37 | 85 | 51.87% |
| Rhodiola_himalensis_XZ_2 | 150953 | 82211 | 25873 | 16996 | 37.76% | 130 | 8 | 37 | 85 | 51.87% |
| Rhodiola_himalensis_XZ_4 | 151853 | 83036 | 25850 | 17117 | 37.69% | 130 | 8 | 37 | 85 | 51.61% |
| Rhodiola_hobsonii_XZ | 151625 | 82885 | 25838 | 17064 | 37.70% | 130 | 8 | 37 | 85 | 51.64% |
| Rhodiola_humilis_var_jialiensis_XZ | 151578 | 82748 | 25872 | 17086 | 37.73% | 130 | 8 | 37 | 85 | 51.66% |
| Rhodiola_kirilowii_HB | 150893 | 82234 | 25835 | 16989 | 37.75% | 130 | 8 | 37 | 85 | 51.91% |
| Rhodiola_kirilowii_SX_2 | 151027 | 82254 | 25872 | 17029 | 37.75% | 130 | 8 | 37 | 85 | 51.86% |
| Rhodiola_kirilowii_XZ_1 | 150997 | 82227 | 25885 | 17000 | 37.76% | 130 | 8 | 37 | 85 | 51.38% |
| Rhodiola_kirilowii_XZ_2 | 150958 | 82198 | 25873 | 17014 | 37.76% | 130 | 8 | 37 | 85 | 51.87% |
| Rhodiola_kirilowii_YN_1 | 151605 | 82854 | 25849 | 17053 | 37.72% | 130 | 8 | 37 | 85 | 51.68% |
| Rhodiola_kirilowii_YN_2 | 151605 | 82854 | 25849 | 17053 | 37.72% | 130 | 8 | 37 | 85 | 51.68% |
| Rhodiola_kirilowii_YN_3 | 151535 | 82892 | 25799 | 17045 | 37.75% | 130 | 8 | 37 | 85 | 51.70% |
| Rhodiola_litwinowii_XJ | 151687 | 82939 | 25849 | 17050 | 37.72% | 130 | 8 | 37 | 85 | 51.66% |
| Rhodiola_nepalica_XZ_1 | 151652 | 82840 | 25867 | 17078 | 37.70% | 130 | 8 | 37 | 85 | 51.62% |
| Rhodiola_nepalica_XZ_2 | 151627 | 82825 | 25863 | 17076 | 37.72% | 130 | 8 | 37 | 85 | 51.64% |
| Rhodiola_nobilis_XZ | 150921 | 82179 | 25873 | 16996 | 37.77% | 130 | 8 | 37 | 85 | 51.73% |
| Rhodiola_prainii_XZ | 151632 | 82828 | 25863 | 17078 | 37.72% | 130 | 8 | 37 | 85 | 51.64% |
| Rhodiola_primuloides_YN_1 | 151334 | 82891 | 25690 | 17063 | 37.68% | 130 | 8 | 37 | 85 | 51.77% |
| Rhodiola_primuloides_YN_2 | 151308 | 82865 | 25690 | 17063 | 37.68% | 130 | 8 | 37 | 85 | 51.78% |
| Rhodiola_primuloides_YN_3 | 151308 | 82865 | 25690 | 17063 | 37.69% | 130 | 8 | 37 | 85 | 51.78% |
| Rhodiola_purpureoviridis_SC | 152014 | 83076 | 25885 | 17168 | 37.71% | 130 | 8 | 37 | 85 | 51.53% |
| Rhodiola_purpureoviridis_XZ_1 | 151768 | 82964 | 25893 | 17018 | 37.69% | 130 | 8 | 37 | 85 | 51.59% |
| Rhodiola_purpureoviridis_XZ_2 | 151957 | 83068 | 25882 | 17125 | 37.70% | 129 | 8 | 37 | 84 | 48.19% |
| Rhodiola_quadrifida_XZ_1 | 151109 | 82206 | 25873 | 17157 | 37.78% | 130 | 8 | 37 | 85 | 51.90% |
| Rhodiola_quadrifida_XZ_2 | 151109 | 82206 | 25873 | 17157 | 37.77% | 130 | 8 | 37 | 85 | 51.83% |
| Rhodiola_quadrifida_XZ_3 | 151768 | 83018 | 25849 | 17052 | 37.70% | 130 | 8 | 37 | 85 | 51.64% |
| Rhodiola_rosea_HB_1 | 152136 | 83330 | 25868 | 17070 | 37.65% | 130 | 8 | 37 | 85 | 51.50% |
| Rhodiola_rosea_HB_2 | 151861 | 83114 | 25853 | 17041 | 37.66% | 130 | 8 | 37 | 85 | 51.56% |
| Rhodiola_rosea_HB_3 | 151861 | 83114 | 25853 | 17041 | 37.66% | 130 | 8 | 37 | 85 | 51.56% |
| Rhodiola_rosea_HB_4 | 151946 | 83149 | 25885 | 17027 | 37.69% | 130 | 8 | 37 | 85 | 51.56% |
| Rhodiola_rosea_JL | 151945 | 83128 | 25882 | 17053 | 37.67% | 130 | 8 | 37 | 85 | 51.59% |
| Rhodiola_rosea_XJ_1 | 151556 | 82775 | 25873 | 17035 | 37.70% | 130 | 8 | 37 | 85 | 51.68% |
| Rhodiola_rosea_XJ_2 | 151729 | 82938 | 25867 | 17057 | 37.68% | 130 | 8 | 37 | 85 | 51.64% |
| Rhodiola_sachalinensis_JL_1 | 151882 | 83089 | 25854 | 17085 | 37.73% | 130 | 8 | 37 | 85 | 51.56% |
| Rhodiola_sacra_XZ_1 | 151625 | 82885 | 25838 | 17064 | 37.70% | 130 | 8 | 37 | 85 | 51.64% |
| Rhodiola_sacra_XZ_2 | 151677 | 82837 | 25882 | 17076 | 37.71% | 130 | 8 | 37 | 85 | 51.62% |
| Rhodiola_sacra_XZ_3 | 151753 | 82933 | 25869 | 17082 | 37.70% | 130 | 8 | 37 | 85 | 51.58% |
| Rhodiola_sacra_XZ_4 | 151640 | 82838 | 25862 | 17078 | 37.69% | 130 | 8 | 37 | 85 | 51.64% |
| Rhodiola_sacra_XZ_5 | 151617 | 82773 | 25872 | 17100 | 37.71% | 130 | 8 | 37 | 85 | 51.66% |
| Rhodiola_sacra_YN | 151647 | 82841 | 25875 | 17056 | 37.71% | 130 | 8 | 37 | 85 | 51.61% |
| Rhodiola_sacra_var_tsuiana_XZ | 151576 | 82759 | 25872 | 17073 | 37.72% | 130 | 8 | 37 | 85 | 51.66% |
| Rhodiola_serrata_XZ_1 | 151830 | 82996 | 25893 | 17048 | 37.69% | 130 | 8 | 37 | 85 | 51.60% |
| Rhodiola_serrata_XZ_2 | 151783 | 82975 | 25884 | 17040 | 37.70% | 130 | 8 | 37 | 85 | 51.61% |
| Rhodiola_sexifolia_XZ | 151573 | 82769 | 25872 | 17060 | 37.72% | 130 | 8 | 37 | 85 | 51.66% |
| Rhodiola_subopposita_XZ | 151007 | 82213 | 25883 | 17028 | 37.75% | 130 | 8 | 37 | 85 | 51.85% |
| Rhodiola_tangutica_XZ_2 | 150975 | 82227 | 25873 | 17002 | 37.77% | 130 | 8 | 37 | 85 | 51.87% |
| Rhodiola_tibetica_XZ | 150968 | 82220 | 25873 | 17002 | 37.79% | 130 | 8 | 37 | 85 | 51.87% |
| Rhodiola_tieghemii_XZ_1 | 151350 | 82593 | 25848 | 17061 | 37.71% | 130 | 8 | 37 | 85 | 51.74% |
| Rhodiola_tieghemii_XZ_2 | 151788 | 82839 | 25941 | 17067 | 37.71% | 130 | 8 | 37 | 85 | 51.57% |
| Rhodiola_wallichiana_XZ | 150932 | 82195 | 25873 | 16991 | 37.76% | 130 | 8 | 37 | 85 | 51.88% |
| Rhodiola_wallichiana_var_cholaensis_XZ_1 | 151009 | 82230 | 25888 | 17003 | 37.76% | 130 | 8 | 37 | 85 | 51.86% |
| Rhodiola_wallichiana_var_cholaensis_XZ_2 | 151022 | 82258 | 25882 | 17000 | 37.76% | 130 | 8 | 37 | 85 | 51.84% |
| Rhodiola_wallichiana_var_cholaensis_YN | 151605 | 82854 | 25849 | 17053 | 37.72% | 130 | 8 | 37 | 85 | 51.68% |
| Rhodiola_yunnanensis_YN_2 | 151634 | 82892 | 25855 | 17032 | 37.74% | 130 | 8 | 37 | 85 | 51.67% |
| Rhodiola_yunnanensis_YN_3 | 151285 | 82689 | 25785 | 17026 | 37.77% | 130 | 8 | 37 | 85 | 51.78% |

**Supplementary Table 3.** Gene annotation of the *Rhodiola* chloroplast genomes.

| Category | Group | Genes |
| --- | --- | --- |
| Photosynthesis related genes | Rubisco | *rbcL* |
|  | Photosystem I | *psaA, psaB, psaC, psaI, psaJ* |
|  | Photosystem II | *psbA, psbB, psbC, psbD, psbE, psbF, psbH, psbI, psbJ, psbK, psbL, psbM, psbN, psbT, psbZ* |
|  | ATP synthase | *atpA, atpB, atpE, atpF^a^, atpH, atpI* |
|  | Cytochrome b/f complex | *petA, petB^a^, petD^a^, petN, petL, petG* |
|  | Cytochrome csynthesis | *ccsA* |
|  | Complex I of chloroplasts | *ndhA^a^, ndhB^a,c^, ndhC, ndhD, ndhE, ndhF, ndhH, ndhG, ndhJ, ndhK, ndhI* |
| Transcription and translation related genes | Transcription | *rpoA, rpoB, rpoC2, rpoC1^a^* |
|  | Ribosomal proteins | *rpl14, rpl16, rpl2^a,c^, rpl20, rpl22, rpl23^c^, rpl32, rpl33, rpl36, rps11, rps12^b,c^, rps14, rps15, rps16^a^, rps18, rps19, rps2, rps3, rps4, rps7^c^, rps8* |
|  | Translation initiation factor | *infA* |
| RNA genes | Ribosomal RNA | *rrn16S^c^, rrn23S^c^, rrn4.5^c^, rrn5^c^* |
|  | Transfer RNA | *trnA-UGC^a,c^, trnC-GCA, trnD-GUC, trnE-UUC, trnF-GAA, trnG-GCC^a^, trnG-UCC, trnH-GUG, trnI-CAU^c^, trnI-GAU^a,c^, trnK-UUU^a^, trnL-CAA^c^, trnL-UAA^a^, trnL-UAG, trnM-CAU, trnN-GUU^c^, trnP-UGG, trnQ-UUG, trnR-ACG^c^, trnR-UCU, trnS-GCU, trnS-GGA, trnS-UGA, trnT-GGU, trnT-UGU, trnV-GAC^c^, trnV-UAC^a^, trnW-CCA, trnY-GUA, trnfM-CAU* |
| Other genes | RNA processing | *matK* |
|  | Carbon metabolism | *cemA* |
|  | Fatty acid synthesis | *accD* |
|  | Proteolysis | *clpP^b^* |
|  | Conserved ORFs | *ycf1, ycf2^c^, ycf3^b^, ycf4* |

^a^genes with one intron, ^b^genes with two introns, ^c^Two gene copies in IRs.

Supplementary Table 4. Genes located on or near the JLB (junction of LSC and IRb) and JSB (junction of SSC and IRb) regions in *Rhodiola* chloroplast genomes.

| Genome_name | JLB_left_gene | on_JLB_gene | JLB_right_gene | JSB_left_gene | on_JSB_gene | JSB_right_gene |
| --- | --- | --- | --- | --- | --- | --- |
| Rhodiola_algida_QH_2 | *rpl2_681,rpl23_376* | *ycf2* |  | *ndhF_136^a^* |  | *rpl32_447* |
| Rhodiola_algida_XZ_1 | *rps3_573,rpl22_77* | *rps19* | *rpl2_294* |  |  | *ndhF_96* |
| Rhodiola_algida_XZ_2 | *rps3_712,rpl22_207* | *rps19* | *rpl2_164* |  | *ndhF* |  |
| Rhodiola_amabilis_XZ_1 | *rps3_709,rpl22_207* | *rps19* | *rpl2_164* |  | *ndhF* |  |
| Rhodiola_amabilis_XZ_2 | *rps3_813,rpl22_311* | *rps19* | *rpl2_60* |  | *ndhF* |  |
| Rhodiola_atuntsuensis_XZ_2 | *rps3_712,rpl22_207* | *rps19* | *rpl2_164* |  | *ndhF* |  |
| Rhodiola_atuntsuensis_XZ_3 | *rps3_725,rpl22_207* | *rps19* | *rpl2_164* |  | *ndhF* |  |
| Rhodiola_atuntsuensis_XZ_4 | *rps3_712,rpl22_207* | *rps19* | *rpl2_164* |  | *ndhF* |  |
| Rhodiola_bupleuroides_XZ_1 | *rps3_708,rpl22_207* | *rps19* | *rpl2_164* |  | *ndhF* |  |
| Rhodiola_bupleuroides_XZ_2 | *rps3_705,rpl22_207* | *rps19* | *rpl2_164* |  | *ndhF* |  |
| Rhodiola_chrysanthemifolia_AG | *rps3_710,rpl22_207* | *rps19* | *rpl2_164* |  | *ndhF* |  |
| Rhodiola_chrysanthemifolia_XZ_1 | *rps3_710,rpl22_207* | *rps19* | *rpl2_164* |  | *ndhF* |  |
| Rhodiola_chrysanthemifolia_XZ_2 | *rps3_710,rpl22_207* | *rps19* | *rpl2_164* |  | *ndhF* |  |
| Rhodiola_crenulata_AG | *rps3_729,rpl22_207* | *rps19* | *rpl2_164* |  | *ndhF* |  |
| Rhodiola_crenulata_SC | *rps3_707,rpl22_207* | *rps19* | *rpl2_164* |  | *ndhF* |  |
| Rhodiola_crenulata_XJ | *rps3_729,rpl22_207* | *rps19* | *rpl2_164* |  | *ndhF* |  |
| Rhodiola_crenulata_XZ_1 | *rps3_729,rpl22_207* | *rps19* | *rpl2_164* |  | *ndhF* |  |
| Rhodiola_crenulata_XZ_2 | *rps3_729,rpl22_207* | *rps19* | *rpl2_164* |  | *ndhF* |  |
| Rhodiola_daochengensis_XZ_1 | *rps3_712,rpl22_207* | *rps19* | *rpl2_164* |  | *ndhF* |  |
| Rhodiola_discolor_SC |  | *ndhF* |  |  |  |  |
| Rhodiola_discolor_XZ |  | *ndhD* | *psaC_358,ndhE_880* |  | *ndhB* |  |
| Rhodiola_dumulosa_NX | *rps3_706,rpl22_207* | *rps19* | *rpl2_164* |  | *ndhF* |  |
| Rhodiola_dumulosa_SX_1 | *rps3_706,rpl22_207* | *rps19* | *rpl2_164* |  | *ndhF* |  |
| Rhodiola_dumulosa_SX_2 | *rps3_706,rpl22_207* | *rps19* | *rpl2_164* |  | *ndhF* |  |
| Rhodiola_fastigiata_AG | *ndhI_802,ndhA_191* |  | *ndhA_990* |  | *ycf2* |  |
| Rhodiola_fastigiata_SC_1 | *rps3_719,rpl22_207* | *rps19* | *rpl2_164* |  | *ndhF* |  |
| Rhodiola_fastigiata_SC_3 | *rps3_707,rpl22_207* | *rps19* | *rpl2_164* |  | *ndhF* |  |
| Rhodiola_fastigiata_XJ_1 | *rps3_712,rpl22_207* | *rps19* | *rpl2_164* |  | *ndhF* |  |
| Rhodiola_fastigiata_XJ_2 | *rps3_708,rpl22_207* | *rps19* | *rpl2_164* |  |  | *ndhF_79* |
| Rhodiola_fastigiata_XZ_1 | *rps3_711,rpl22_207* | *rps19* | *rpl2_164* |  | *ndhF* |  |
| Rhodiola_fastigiata_XZ_2 |  | *ndhF* |  |  |  |  |
| Rhodiola_fastigiata_YN_1 | *rps3_708,rpl22_207* | *rps19* | *rpl2_164* |  | *ndhF* |  |
| Rhodiola_forrestii_XZ | *rps3_713,rpl22_207* | *rps19* | *rpl2_164* |  | *ndhF* |  |
| Rhodiola_forrestii_YN | *rps3_708,rpl22_207* | *rps19* | *rpl2_164* |  | *ndhF* |  |
| Rhodiola_himalensis_XZ_1 | *rps3_712,rpl22_207* | *rps19* | *rpl2_164* |  | *ndhF* |  |
| Rhodiola_himalensis_XZ_2 | *rps3_714,rpl22_207* | *rps19* | *rpl2_164* |  | *ndhF* |  |
| Rhodiola_himalensis_XZ_4 | *rps3_708,rpl22_207* | *rps19* | *rpl2_164* |  | *ndhF* |  |
| Rhodiola_hobsonii_XZ | *rps3_710,rpl22_207* | *rps19* | *rpl2_164* |  | *ndhF* |  |
| Rhodiola_humilis_var_jialiensis_XZ | *rps3_710,rpl22_207* | *rps19* | *rpl2_164* |  | *ndhF* |  |
| Rhodiola_kirilowii_HB | *rps3_713,rpl22_207* | *rps19* | *rpl2_164* |  | *ndhF* |  |
| Rhodiola_kirilowii_SX_2 | *rps3_713,rpl22_209* | *rps19* | *rpl2_162* |  | *ndhF* |  |
| Rhodiola_kirilowii_XZ_1 | *rps3_707,rpl22_207* | *rps19* | *rpl2_164* |  |  | *ndhF_710* |
| Rhodiola_kirilowii_XZ_2 | *rps3_712,rpl22_207* | *rps19* | *rpl2_164* |  | *ndhF* |  |
| Rhodiola_kirilowii_YN_1 | *rps3_708,rpl22_207* | *rps19* | *rpl2_164* |  | *ndhF* |  |
| Rhodiola_kirilowii_YN_2 | *rps3_708,rpl22_207* | *rps19* | *rpl2_164* |  | *ndhF* |  |
| Rhodiola_kirilowii_YN_3 | *rps3_708,rpl22_207* | *rps19* | *rpl2_164* |  | *ndhF* |  |
| Rhodiola_litwinowii_XJ | *rps3_708,rpl22_207* | *rps19* | *rpl2_164* |  | *ndhF* |  |
| Rhodiola_nepalica_XZ_1 | *rps3_709,rpl22_207* | *rps19* | *rpl2_164* |  | *ndhF* |  |
| Rhodiola_nepalica_XZ_2 | *rps3_709,rpl22_207* | *rps19* | *rpl2_164* |  | *ndhF* |  |
| Rhodiola_nobilis_XZ | *rps3_707,rpl22_207* | *rps19* | *rpl2_164* |  | *ndhF* |  |
| Rhodiola_prainii_XZ | *rps3_709,rpl22_207* | *rps19* | *rpl2_164* |  | *ndhF* |  |
| Rhodiola_primuloides_YN_1 | *rps3_707,rpl22_207* | *rps19* | *rpl2_164* |  | *ndhF* |  |
| Rhodiola_primuloides_YN_2 | *rps3_707,rpl22_207* | *rps19* | *rpl2_164* |  | *ndhF* |  |
| Rhodiola_primuloides_YN_3 | *rps3_707,rpl22_207* | *rps19* | *rpl2_164* |  | *ndhF* |  |
| Rhodiola_purpureoviridis_SC |  | *ycf1* |  | *ycf2_91* |  | *rpl23_238,rpl2_543* |
| Rhodiola_purpureoviridis_XZ_1 | *rps3_705,rpl22_207* | *rps19* | *rpl2_164* |  | *ndhF* |  |
| Rhodiola_purpureoviridis_XZ_2 | *rps3_708,rpl22_207* | *rps19* | *rpl2_164* |  | *ndhF* |  |
| Rhodiola_quadrifida_XZ_1 |  | *ycf1* |  | *rpl2_19* |  |  |
| Rhodiola_quadrifida_XZ_2 |  | *ycf1* |  | *rpl2_165* |  |  |
| Rhodiola_quadrifida_XZ_3 | *rps3_716,rpl22_207* | *rps19* | *rpl2_164* |  | *ndhF* |  |
| Rhodiola_rosea_HB_1 | *rps3_706,rpl22_207* | *rps19* | *rpl2_164* |  | *ndhF* |  |
| Rhodiola_rosea_HB_2 | *rps3_706,rpl22_207* | *rps19* | *rpl2_164* |  | *ndhF* |  |
| Rhodiola_rosea_HB_3 | *rps3_706,rpl22_207* | *rps19* | *rpl2_164* |  | *ndhF* |  |
| Rhodiola_rosea_HB_4 | *rps3_689,rpl22_207* | *rps19* | *rpl2_164* |  | *ndhF* |  |
| Rhodiola_rosea_JL | *rps3_719,rpl22_207* | *rps19* | *rpl2_164* |  | *ndhF* |  |
| Rhodiola_rosea_XJ_1 | *rps3_652,rpl22_207* | *rps19* | *rpl2_164* |  | *ndhF* |  |
| Rhodiola_rosea_XJ_2 | *rps3_710,rpl22_211* | *rps19* | *rpl2_160* |  | *ndhF* |  |
| Rhodiola_sachalinensis_JL_1 | *rps3_713,rpl22_207* | *rps19* | *rpl2_164* |  | *ndhF* |  |
| Rhodiola_sacra_XZ_1 | *rps3_710,rpl22_207* | *rps19* | *rpl2_164* |  | *ndhF* |  |
| Rhodiola_sacra_XZ_2 | *rps3_709,rpl22_207* | *rps19* | *rpl2_164* |  | *ndhF* |  |
| Rhodiola_sacra_XZ_3 | *rps3_709,rpl22_207* | *rps19* | *rpl2_164* |  | *ndhF* |  |
| Rhodiola_sacra_XZ_4 | *rps3_709,rpl22_207* | *rps19* | *rpl2_164* |  | *ndhF* |  |
| Rhodiola_sacra_XZ_5 | *rps3_709,rpl22_207* | *rps19* | *rpl2_164* |  | *ndhF* |  |
| Rhodiola_sacra_YN | *rps3_711,rpl22_207* | *rps19* | *rpl2_164* |  | *ndhF* |  |
| Rhodiola_sacra_var_tsuiana_XZ | *rps3_710,rpl22_207* | *rps19* | *rpl2_164* |  | *ndhF* |  |
| Rhodiola_serrata_XZ_1 | *rps3_705,rpl22_207* | *rps19* | *rpl2_164* |  | *ndhF* |  |
| Rhodiola_serrata_XZ_2 | *rps3_705,rpl22_207* | *rps19* | *rpl2_164* |  | *ndhF* |  |
| Rhodiola_sexifolia_XZ | *rps3_710,rpl22_207* | *rps19* | *rpl2_164* |  | *ndhF* |  |
| Rhodiola_subopposita_XZ | *rps3_696,rpl22_207* | *rps19* | *rpl2_164* |  | *ndhF* |  |
| Rhodiola_tangutica_XZ_2 | *rps3_712,rpl22_207* | *rps19* | *rpl2_164* |  | *ndhF* |  |
| Rhodiola_tibetica_XZ | *rps3_708,rpl22_207* | *rps19* | *rpl2_164* |  | *ndhF* |  |
| Rhodiola_tieghemii_XZ_1 | *rps3_709,rpl22_207* | *rps19* | *rpl2_164* |  | *ndhF* |  |
| Rhodiola_tieghemii_XZ_2 | *rps3_711,rpl22_207* | *rps19* | *rpl2_164* |  | *ndhF* |  |
| Rhodiola_wallichiana_XZ | *rps3_711,rpl22_207* | *rps19* | *rpl2_164* |  | *ndhF* |  |
| Rhodiola_wallichiana_var_cholaensis_XZ_1 | *rps3_710,rpl22_207* | *rps19* | *rpl2_164* |  | *ndhF* |  |
| Rhodiola_wallichiana_var_cholaensis_XZ_2 | *rps3_712,rpl22_207* | *rps19* | *rpl2_164* |  | *ndhF* |  |
| Rhodiola_wallichiana_var_cholaensis_YN | *rps3_708,rpl22_207* | *rps19* | *rpl2_164* |  | *ndhF* |  |
| Rhodiola_yunnanensis_YN_2 | *rps3_707,rpl22_207* | *rps19* | *rpl2_164* |  | *ndhF* |  |
| Rhodiola_yunnanensis_YN_3 | *rps3_719,rpl22_207* | *rps19* | *rpl2_164* |  | *ndhF* |  |

* “_num” means the distance of the between gene and junction site. For example, a (“ndnF_136”) means that ndhF is 136bp to the right of JSB left.

**Supplementary Table 5.** Genes located on or near the JSA (junction of SSC and IRa) and JLA (junction of LSC and IRa) regions in *Rhodiola* chloroplast genomes.

| Genome_name | JSA_left_gene | on_JSA_gene | JSA_right_gene | JLA_left_gene | on_JLA_gene | JLA_right_gene |
| --- | --- | --- | --- | --- | --- | --- |
| Rhodiola_algida_QH_2 |  |  |  | *rpl2_165* |  |  |
| Rhodiola_algida_XZ_1 |  | *ycf1* |  | *rpl2_35* |  |  |
| Rhodiola_algida_XZ_2 |  | *ycf1* |  | *rpl2_165* |  |  |
| Rhodiola_amabilis_XZ_1 |  | *ycf1* |  | *rpl2_165* |  |  |
| Rhodiola_amabilis_XZ_2 |  | *ycf1* |  | *rpl2_270* |  |  |
| Rhodiola_atuntsuensis_XZ_2 |  | *ycf1* |  | *rpl2_165* |  |  |
| Rhodiola_atuntsuensis_XZ_3 |  | *ycf1* |  | *rpl2_165* |  |  |
| Rhodiola_atuntsuensis_XZ_4 |  | *ycf1* |  | *rpl2_165* |  |  |
| Rhodiola_bupleuroides_XZ_1 |  | *ycf1* |  | *rpl2_165* |  |  |
| Rhodiola_bupleuroides_XZ_2 |  | *ycf1* |  | *rpl2_165* |  |  |
| Rhodiola_chrysanthemifolia_AG |  | *ycf1* |  | *rpl2_165* |  |  |
| Rhodiola_chrysanthemifolia_XZ_1 |  | *ycf1* |  | *rpl2_165* |  |  |
| Rhodiola_chrysanthemifolia_XZ_2 |  | *ycf1* |  | *rpl2_165* |  |  |
| Rhodiola_crenulata_AG |  | *ycf1* |  | *rpl2_165* |  |  |
| Rhodiola_crenulata_SC |  | *ycf1* |  | *rpl2_165* |  |  |
| Rhodiola_crenulata_XJ |  | *ycf1* |  | *rpl2_165* |  |  |
| Rhodiola_crenulata_XZ_1 |  | *ycf1* |  | *rpl2_165* |  |  |
| Rhodiola_crenulata_XZ_2 |  | *ycf1* |  | *rpl2_165* |  |  |
| Rhodiola_daochengensis_XZ_1 |  | *ycf1* |  | *rpl2_165* |  |  |
| Rhodiola_discolor_SC |  |  | *psbA_302* |  | *rpoB* |  |
| Rhodiola_discolor_XZ | *rps16_219* |  |  |  |  |  |
| Rhodiola_dumulosa_NX |  | *ycf1* |  | *rpl2_165* |  |  |
| Rhodiola_dumulosa_SX_1 |  | *ycf1* |  | *rpl2_165* |  |  |
| Rhodiola_dumulosa_SX_2 |  | *ycf1* |  | *rpl2_165* |  |  |
| Rhodiola_fastigiata_AG |  |  | *atpA_161* | *psbC_516* |  | *psbZ_65* |
| Rhodiola_fastigiata_SC_1 |  | *ycf1* |  | *rpl2_165* |  |  |
| Rhodiola_fastigiata_SC_3 |  | *ycf1* |  | *rpl2_165* |  |  |
| Rhodiola_fastigiata_XJ_1 |  | *ycf1* |  | *rpl2_165* |  |  |
| Rhodiola_fastigiata_XJ_2 |  | *ycf1* |  | *rpl2_165* |  |  |
| Rhodiola_fastigiata_XZ_1 |  | *ycf1* |  | *rpl2_165* |  |  |
| Rhodiola_fastigiata_XZ_2 |  |  | *psbA_327* |  | *rpoB* |  |
| Rhodiola_fastigiata_YN_1 |  | *ycf1* |  | *rpl2_165* |  |  |
| Rhodiola_forrestii_XZ |  | *ycf1* |  | *rpl2_165* |  |  |
| Rhodiola_forrestii_YN |  | *ycf1* |  | *rpl2_165* |  |  |
| Rhodiola_himalensis_XZ_1 |  | *ycf1* |  | *rpl2_165* |  |  |
| Rhodiola_himalensis_XZ_2 |  | *ycf1* |  | *rpl2_165* |  |  |
| Rhodiola_himalensis_XZ_4 |  | *ycf1* |  | *rpl2_165* |  |  |
| Rhodiola_hobsonii_XZ |  | *ycf1* |  | *rpl2_165* |  |  |
| Rhodiola_humilis_var_jialiensis_XZ |  | *ycf1* |  | *rpl2_165* |  |  |
| Rhodiola_kirilowii_HB |  | *ycf1* |  | *rpl2_165* |  |  |
| Rhodiola_kirilowii_SX_2 |  | *ycf1* |  | *rpl2_165* |  |  |
| Rhodiola_kirilowii_XZ_1 |  | *ycf1* |  | *rpl2_165* |  |  |
| Rhodiola_kirilowii_XZ_2 |  | *ycf1* |  | *rpl2_165* |  |  |
| Rhodiola_kirilowii_YN_1 |  | *ycf1* |  | *rpl2_165* |  |  |
| Rhodiola_kirilowii_YN_2 |  | *ycf1* |  | *rpl2_165* |  |  |
| Rhodiola_kirilowii_YN_3 |  | *ycf1* |  | *rpl2_165* |  |  |
| Rhodiola_litwinowii_XJ |  | *ycf1* |  | *rpl2_165* |  |  |
| Rhodiola_nepalica_XZ_1 |  | *ycf1* |  | *rpl2_165* |  |  |
| Rhodiola_nepalica_XZ_2 |  | *ycf1* |  | *rpl2_165* |  |  |
| Rhodiola_nobilis_XZ |  | *ycf1* |  | *rpl2_165* |  |  |
| Rhodiola_prainii_XZ |  | *ycf1* |  | *rpl2_165* |  |  |
| Rhodiola_primuloides_YN_1 |  | *ycf1* |  | *rpl2_165* |  |  |
| Rhodiola_primuloides_YN_2 |  | *ycf1* |  | *rpl2_165* |  |  |
| Rhodiola_primuloides_YN_3 |  | *ycf1* |  | *rpl2_165* |  |  |
| Rhodiola_purpureoviridis_SC | *atpH_973* | *atpI* | *rps2_656* | *psaB_694* | *psaA* |  |
| Rhodiola_purpureoviridis_XZ_1 |  | *ycf1* |  | *rpl2_165* |  |  |
| Rhodiola_purpureoviridis_XZ_2 |  |  |  | *rpl2_165* |  |  |
| Rhodiola_quadrifida_XZ_1 |  | *rpoC2* |  | *ycf3_526* |  | *ycf3_218* |
| Rhodiola_quadrifida_XZ_2 |  | *rpoC2* |  | *ycf3_672* |  | *ycf3_994,ycf3_72* |
| Rhodiola_quadrifida_XZ_3 |  | *ycf1* |  | *rpl2_165* |  |  |
| Rhodiola_rosea_HB_1 |  | *ycf1* |  | *rpl2_165* |  |  |
| Rhodiola_rosea_HB_2 |  | *ycf1* |  | *rpl2_165* |  |  |
| Rhodiola_rosea_HB_3 |  | *ycf1* |  | *rpl2_165* |  |  |
| Rhodiola_rosea_HB_4 |  | *ycf1* |  | *rpl2_165* |  |  |
| Rhodiola_rosea_JL |  | *ycf1* |  | *rpl2_165* |  |  |
| Rhodiola_rosea_XJ_1 |  | *ycf1* |  | *rpl2_165* |  |  |
| Rhodiola_rosea_XJ_2 |  | *ycf1* |  | *rpl2_165* |  |  |
| Rhodiola_sachalinensis_JL_1 |  | *ycf1* |  | *rpl2_165* |  |  |
| Rhodiola_sacra_XZ_1 |  | *ycf1* |  | *rpl2_165* |  |  |
| Rhodiola_sacra_XZ_2 |  | *ycf1* |  | *rpl2_165* |  |  |
| Rhodiola_sacra_XZ_3 |  | *ycf1* |  | *rpl2_165* |  |  |
| Rhodiola_sacra_XZ_4 |  | *ycf1* |  | *rpl2_165* |  |  |
| Rhodiola_sacra_XZ_5 |  | *ycf1* |  | *rpl2_165* |  |  |
| Rhodiola_sacra_YN |  | *ycf1* |  | *rpl2_165* |  |  |
| Rhodiola_sacra_var_tsuiana_XZ |  | *ycf1* |  | *rpl2_165* |  |  |
| Rhodiola_serrata_XZ_1 |  | *ycf1* |  | *rpl2_165* |  |  |
| Rhodiola_serrata_XZ_2 |  | *ycf1* |  | *rpl2_165* |  |  |
| Rhodiola_sexifolia_XZ |  | *ycf1* |  | *rpl2_165* |  |  |
| Rhodiola_subopposita_XZ |  | *ycf1* |  | *rpl2_165* |  |  |
| Rhodiola_tangutica_XZ_2 |  | *ycf1* |  | *rpl2_165* |  |  |
| Rhodiola_tibetica_XZ |  | *ycf1* |  | *rpl2_165* |  |  |
| Rhodiola_tieghemii_XZ_1 |  | *ycf1* |  | *rpl2_165* |  |  |
| Rhodiola_tieghemii_XZ_2 |  | *ycf1* |  | *rpl2_165* |  |  |
| Rhodiola_wallichiana_XZ |  | *ycf1* |  | *rpl2_165* |  |  |
| Rhodiola_wallichiana_var_cholaensis_XZ_1 |  | *ycf1* |  | *rpl2_165* |  |  |
| Rhodiola_wallichiana_var_cholaensis_XZ_2 |  | *ycf1* |  | *rpl2_165* |  |  |
| Rhodiola_wallichiana_var_cholaensis_YN |  | *ycf1* |  | *rpl2_165* |  |  |
| Rhodiola_yunnanensis_YN_2 |  | *ycf1* |  | *rpl2_165* |  |  |
| Rhodiola_yunnanensis_YN_3 |  | *ycf1* |  | *rpl2_165* |  |  |

* “_num” has the same meaning as Supplementary Table 4.

**Supplementary Table 6.** Numbers and types of simple sequence repeats (SSRs) in *Rhodiola* chloroplast genomes.

| Genome_name | mono | di | tri | tetra | penta | hexa | SSR_seq |
| --- | --- | --- | --- | --- | --- | --- | --- |
| Rhodiola_algida_QH_2 | 38 | 8 | 1 | 5 | 0 | 0 | ['A', 'T', 'AT', 'TA', 'TTG', 'AATA', 'ATTT', 'GATA', 'GTCT', 'GTTT'] |
| Rhodiola_algida_XZ_1 | 41 | 9 | 0 | 4 | 0 | 0 | ['A', 'T', 'AT', 'TA', 'AATA', 'GATA', 'GTCT', 'GTTT'] |
| Rhodiola_algida_XZ_2 | 43 | 8 | 0 | 4 | 0 | 0 | ['A', 'T', 'AT', 'TA', 'AATA', 'GATA', 'GTCT', 'GTTT'] |
| Rhodiola_amabilis_XZ_1 | 43 | 7 | 0 | 3 | 0 | 0 | ['A', 'T', 'AT', 'TA', 'AATA', 'GATA', 'GTCT'] |
| Rhodiola_amabilis_XZ_2 | 40 | 6 | 0 | 3 | 1 | 0 | ['A', 'T', 'AT', 'TA', 'AATA', 'GATA', 'GTCT', 'ATAAA'] |
| Rhodiola_atuntsuensis_XZ_2 | 43 | 8 | 0 | 4 | 0 | 0 | ['A', 'T', 'AT', 'TA', 'AATA', 'GATA', 'GTCT', 'GTTT'] |
| Rhodiola_atuntsuensis_XZ_3 | 40 | 8 | 0 | 4 | 0 | 0 | ['A', 'T', 'AT', 'TA', 'AATA', 'GATA', 'GTCT', 'GTTT'] |
| Rhodiola_bupleuroides_XZ_1 | 34 | 6 | 1 | 6 | 2 | 0 | ['A', 'T', 'AT', 'TA', 'AATA', 'GATA', 'GTCT', 'GTTT', 'ATTCTA'] |
| Rhodiola_bupleuroides_XZ_2 | 35 | 6 | 1 | 4 | 0 | 0 | ['A', 'T', 'AT', 'TA', 'TTG', 'AATA', 'ATTT', 'GATA', 'GTCT', 'GTTT', 'TATT', 'ATATA', 'TTCTA'] |
| Rhodiola_chrysanthemifolia_AG | 37 | 6 | 0 | 4 | 0 | 0 | ['A', 'T', 'AT', 'TA', 'TTG', 'AATA', 'GATA', 'GTCT', 'GTTT'] |
| Rhodiola_chrysanthemifolia_XZ_1 | 37 | 6 | 0 | 4 | 0 | 0 | ['A', 'T', 'AT', 'TA', 'AATA', 'ATTT', 'GATA', 'GTCT'] |
| Rhodiola_chrysanthemifolia_XZ_2 | 37 | 6 | 0 | 4 | 0 | 0 | ['A', 'T', 'AT', 'TA', 'AATA', 'ATTT', 'GATA', 'GTCT'] |
| Rhodiola_crenulata_AG | 33 | 9 | 0 | 4 | 0 | 0 | ['A', 'T', 'AT', 'TA', 'AATA', 'ATTT', 'GATA', 'GTCT'] |
| Rhodiola_crenulata_SC | 38 | 9 | 0 | 4 | 0 | 0 | ['A', 'T', 'AT', 'TA', 'AATA', 'GATA', 'GTCT', 'GTTT'] |
| Rhodiola_crenulata_XJ | 34 | 9 | 0 | 4 | 0 | 0 | ['A', 'T', 'AT', 'TA', 'AATA', 'GATA', 'GTCT', 'GTTT'] |
| Rhodiola_crenulata_XZ_1 | 42 | 8 | 0 | 4 | 0 | 0 | ['A', 'T', 'AT', 'TA', 'AATA', 'GATA', 'GTCT', 'GTTT'] |
| Rhodiola_crenulata_XZ_2 | 39 | 8 | 0 | 4 | 0 | 0 | ['A', 'T', 'AT', 'TA', 'AATA', 'GATA', 'GTCT', 'GTTT'] |
| Rhodiola_daochengensis_XZ_1 | 36 | 8 | 0 | 4 | 0 | 0 | ['A', 'T', 'AT', 'TA', 'AATA', 'GATA', 'GTCT', 'GTTT'] |
| Rhodiola_discolor_SC | 37 | 6 | 1 | 4 | 0 | 0 | ['A', 'T', 'AT', 'TA', 'AATA', 'GATA', 'GTCT', 'GTTT'] |
| Rhodiola_dumulosa_NX | 40 | 8 | 0 | 4 | 0 | 0 | ['A', 'T', 'AT', 'TA', 'TTG', 'AATA', 'GATA', 'GTCT', 'GTTT'] |
| Rhodiola_dumulosa_SX_1 | 41 | 8 | 0 | 4 | 0 | 0 | ['A', 'T', 'AT', 'TA', 'AATA', 'GATA', 'GTCT', 'GTTT', 'TTAGAA'] |
| Rhodiola_dumulosa_SX_2 | 39 | 8 | 0 | 4 | 0 | 0 | ['A', 'T', 'AT', 'TA', 'AATA', 'GATA', 'GTCT', 'TCTG'] |
| Rhodiola_fastigiata_AG | 40 | 8 | 0 | 4 | 0 | 0 | ['A', 'T', 'AT', 'TA', 'AATA', 'GATA', 'GTCT', 'TCTG'] |
| Rhodiola_fastigiata_SC_1 | 42 | 8 | 0 | 5 | 0 | 0 | ['A', 'T', 'AT', 'TA', 'AATA', 'GATA', 'GTCT', 'TCTG'] |
| Rhodiola_fastigiata_SC_3 | 37 | 6 | 0 | 4 | 0 | 0 | ['A', 'T', 'AT', 'TA', 'AATA', 'GATA', 'GTCT', 'GTTT'] |
| Rhodiola_fastigiata_XJ_1 | 41 | 8 | 0 | 4 | 0 | 0 | ['A', 'T', 'AT', 'TA', 'AATA', 'GATA', 'GTCT', 'GTTT', 'TTAA'] |
| Rhodiola_fastigiata_XJ_2 | 44 | 8 | 0 | 4 | 0 | 0 | ['A', 'C', 'T', 'AT', 'TA', 'AATA', 'GATA', 'GTCT', 'GTTT'] |
| Rhodiola_fastigiata_XZ_1 | 37 | 7 | 0 | 4 | 0 | 0 | ['A', 'T', 'AT', 'TA', 'AATA', 'GATA', 'GTCT', 'GTTT'] |
| Rhodiola_fastigiata_XZ_2 | 37 | 8 | 0 | 4 | 0 | 0 | ['A', 'C', 'T', 'AT', 'TA', 'AATA', 'GATA', 'GTCT', 'GTTT'] |
| Rhodiola_fastigiata_YN_1 | 43 | 8 | 0 | 5 | 0 | 0 | ['A', 'T', 'AT', 'TA', 'AATA', 'GATA', 'GTCT', 'GTTT'] |
| Rhodiola_forrestii_XZ | 43 | 7 | 0 | 4 | 0 | 0 | ['A', 'T', 'AT', 'TA', 'AATA', 'GATA', 'GTCT', 'GTTT'] |
| Rhodiola_forrestii_YN | 37 | 7 | 1 | 5 | 0 | 0 | ['A', 'T', 'AT', 'TA', 'AAAG', 'AATA', 'GATA', 'GTCT', 'GTTT'] |
| Rhodiola_himalensis_XZ_2 | 38 | 8 | 0 | 4 | 0 | 0 | ['A', 'G', 'T', 'AT', 'TA', 'AATA', 'GATA', 'GTCT', 'TTAA'] |
| Rhodiola_himalensis_XZ_4 | 43 | 8 | 1 | 4 | 0 | 0 | ['A', 'T', 'AT', 'TA', 'GTT', 'AATA', 'ATTT', 'GATA', 'GTCT', 'GTTT'] |
| Rhodiola_hobsonii_XZ | 36 | 6 | 0 | 3 | 0 | 0 | ['A', 'T', 'AT', 'TA', 'AATA', 'ATAA', 'GATA', 'GTCT', 'GTTT', 'GTACTA', 'TAGTAC'] |
| Rhodiola_humilis_var_jialiensis_XZ | 42 | 6 | 0 | 4 | 0 | 0 | ['A', 'T', 'AT', 'TA', 'AATA', 'GATA', 'GTCT', 'GTTT'] |
| Rhodiola_kirilowii_HB | 40 | 8 | 0 | 4 | 0 | 0 | ['A', 'T', 'AT', 'TA', 'TTA', 'AATA', 'GATA', 'GTCT', 'GTTT'] |
| Rhodiola_kirilowii_SX_2 | 41 | 8 | 0 | 4 | 0 | 0 | ['A', 'T', 'AT', 'TA', 'AATA', 'GATA', 'GTCT'] |
| Rhodiola_kirilowii_XZ_2 | 40 | 8 | 0 | 4 | 0 | 0 | ['A', 'T', 'AT', 'TA', 'AATA', 'ATTT', 'GATA', 'GTCT'] |
| Rhodiola_kirilowii_YN_1 | 38 | 7 | 0 | 4 | 1 | 0 | ['A', 'T', 'AT', 'TA', 'AATA', 'GATA', 'GTCT', 'GTTT'] |
| Rhodiola_kirilowii_YN_2 | 38 | 7 | 0 | 4 | 1 | 0 | ['A', 'T', 'AT', 'TA', 'AATA', 'GATA', 'GTCT', 'GTTT'] |
| Rhodiola_kirilowii_YN_3 | 39 | 7 | 1 | 4 | 0 | 0 | ['A', 'T', 'AT', 'TA', 'AATA', 'GATA', 'GTCT', 'GTTT', 'GTACTA', 'TAGTAC'] |
| Rhodiola_litwinowii_XJ | 40 | 8 | 0 | 4 | 1 | 0 | ['A', 'C', 'T', 'AT', 'TA', 'AATA', 'GATA', 'GTCT', 'GTTT'] |
| Rhodiola_nepalica_XZ_1 | 40 | 5 | 0 | 3 | 1 | 0 | ['A', 'T', 'AT', 'TA', 'AATA', 'GATA', 'GTCT', 'GTTT', 'TCTGG'] |
| Rhodiola_nepalica_XZ_2 | 40 | 6 | 0 | 3 | 0 | 0 | ['A', 'T', 'AT', 'TA', 'AATA', 'GATA', 'GTCT', 'GTTT', 'TCTGG'] |
| Rhodiola_nobilis_XZ | 38 | 8 | 0 | 4 | 0 | 0 | ['A', 'T', 'AT', 'TA', 'GTT', 'AATA', 'GATA', 'GTCT', 'GTTT'] |
| Rhodiola_prainii_XZ | 42 | 6 | 0 | 3 | 0 | 0 | ['A', 'T', 'AT', 'TA', 'AATA', 'GATA', 'GTCT', 'GTTT', 'ATATA'] |
| Rhodiola_primuloides_YN_1 | 39 | 7 | 0 | 3 | 0 | 0 | ['A', 'T', 'AT', 'TA', 'AATA', 'GATA', 'GTCT', 'GTTTT'] |
| Rhodiola_primuloides_YN_2 | 39 | 7 | 0 | 3 | 0 | 0 | ['A', 'C', 'T', 'AT', 'TA', 'AATA', 'GATA', 'GTCT'] |
| Rhodiola_primuloides_YN_3 | 39 | 7 | 0 | 3 | 0 | 0 | ['A', 'C', 'T', 'AT', 'TA', 'AATA', 'GATA', 'GTCT', 'GTTT'] |
| Rhodiola_purpureoviridis_SC | 40 | 6 | 1 | 4 | 0 | 0 | ['A', 'C', 'T', 'AT', 'TA', 'AATA', 'GATA', 'GTCT'] |
| Rhodiola_purpureoviridis_XZ_1 | 35 | 7 | 0 | 5 | 0 | 0 | ['A', 'T', 'AT', 'TA', 'AATA', 'GATA', 'GTCT'] |
| Rhodiola_purpureoviridis_XZ_2 | 44 | 7 | 1 | 4 | 0 | 0 | ['A', 'T', 'AT', 'TA', 'AATA', 'GATA', 'GTCT'] |
| Rhodiola_quadrifida_XZ_1 | 40 | 8 | 1 | 4 | 1 | 0 | ['A', 'T', 'AT', 'TA', 'AATA', 'GATA', 'GTCT'] |
| Rhodiola_quadrifida_XZ_2 | 40 | 8 | 1 | 4 | 1 | 0 | ['A', 'T', 'AT', 'TA', 'TTG', 'AATA', 'GATA', 'GTCT', 'GTTT'] |
| Rhodiola_quadrifida_XZ_3 | 44 | 8 | 0 | 4 | 1 | 0 | ['A', 'T', 'AT', 'TA', 'AATA', 'GATA', 'GTCT', 'GTTT', 'TTTC'] |
| Rhodiola_rosea_HB_1 | 36 | 7 | 1 | 4 | 0 | 0 | ['A', 'T', 'AT', 'TA', 'TTG', 'AATA', 'GATA', 'GTCT', 'GTTT'] |
| Rhodiola_rosea_HB_2 | 40 | 9 | 0 | 4 | 1 | 0 | ['A', 'T', 'AT', 'TA', 'ATT', 'AATA', 'GATA', 'GTCT', 'GTTT', 'TCTTA'] |
| Rhodiola_rosea_HB_3 | 40 | 9 | 0 | 4 | 1 | 0 | ['A', 'T', 'AT', 'TA', 'ATT', 'AATA', 'GATA', 'GTCT', 'GTTT', 'TCTTA'] |
| Rhodiola_rosea_HB_4 | 39 | 7 | 0 | 5 | 0 | 0 | ['A', 'T', 'AT', 'TA', 'AATA', 'GATA', 'GTCT', 'GTTT', 'TCTGG'] |
| Rhodiola_rosea_XJ_1 | 40 | 7 | 1 | 4 | 0 | 0 | ['A', 'T', 'AT', 'TA', 'AAT', 'AATA', 'GATA', 'GTCT', 'GTTT'] |
| Rhodiola_rosea_XJ_2 | 43 | 7 | 0 | 4 | 0 | 0 | ['A', 'T', 'AT', 'TA', 'AATA', 'GATA', 'GTCT', 'GTTT', 'ATAAA'] |
| Rhodiola_sachalinensis_JL_1 | 39 | 8 | 0 | 3 | 0 | 0 | ['A', 'T', 'AT', 'TA', 'AATA', 'GATA', 'GTCT', 'GTTT', 'ATAAA'] |
| Rhodiola_sacra_var_tsuiana_XZ | 36 | 4 | 0 | 3 | 0 | 0 | ['A', 'T', 'AT', 'TA', 'AATA', 'GATA', 'GTCT', 'GTTT', 'TATT'] |
| Rhodiola_sacra_XZ_1 | 36 | 6 | 0 | 3 | 0 | 0 | ['A', 'T', 'AT', 'TA', 'AAT', 'AATA', 'GATA', 'GTCT', 'GTTT', 'AATTAG', 'TTTACT'] |
| Rhodiola_sacra_XZ_2 | 42 | 6 | 0 | 3 | 0 | 0 | ['A', 'T', 'AT', 'TA', 'AAT', 'AATA', 'GATA', 'GTCT', 'GTTT'] |
| Rhodiola_sacra_XZ_3 | 42 | 5 | 0 | 3 | 1 | 0 | ['A', 'T', 'AT', 'TA', 'AATA', 'GATA', 'GTCT', 'GTTT'] |
| Rhodiola_sacra_XZ_4 | 43 | 5 | 0 | 4 | 1 | 0 | ['A', 'T', 'AT', 'TA', 'AATA', 'GATA', 'GTCT'] |
| Rhodiola_sacra_XZ_5 | 41 | 5 | 0 | 4 | 1 | 0 | ['A', 'T', 'AT', 'TA', 'AATA', 'GATA', 'GTCT'] |
| Rhodiola_serrata_XZ_1 | 39 | 7 | 0 | 4 | 0 | 0 | ['A', 'T', 'AT', 'TA', 'AATA', 'GATA', 'GTCT'] |
| Rhodiola_serrata_XZ_2 | 35 | 7 | 0 | 5 | 0 | 0 | ['A', 'C', 'T', 'AT', 'TA', 'AATA', 'GATA', 'GTCT'] |
| Rhodiola_sexifolia_XZ | 36 | 4 | 0 | 4 | 0 | 0 | ['A', 'T', 'AT', 'TA', 'AATA', 'GATA', 'GTCT', 'GTTTT'] |
| Rhodiola_subopposita_XZ | 43 | 7 | 0 | 4 | 0 | 0 | ['A', 'T', 'AT', 'TA', 'AATA', 'ATTT', 'GATA', 'GTCT', 'GTTTT'] |
| Rhodiola_tangutica_XZ_2 | 40 | 8 | 0 | 4 | 0 | 0 | ['A', 'T', 'AT', 'TA', 'AATA', 'ATTT', 'GATA', 'GTCT', 'GTTTT'] |
| Rhodiola_tibetica_XZ | 46 | 7 | 1 | 4 | 1 | 0 | ['A', 'T', 'AT', 'TA', 'AATA', 'ATTT', 'GATA', 'GTCT', 'GTTTT', 'TACTTA', 'TATAAG'] |
| Rhodiola_tieghemii_XZ_1 | 38 | 6 | 0 | 4 | 0 | 0 | ['A', 'C', 'T', 'AT', 'TA', 'AATA', 'GATA', 'GTCT', 'GTTT'] |
| Rhodiola_tieghemii_XZ_2 | 40 | 6 | 0 | 4 | 0 | 0 | ['A', 'T', 'AT', 'TA', 'AATA', 'GATA', 'GTCT', 'GTTT', 'TTTC'] |
| Rhodiola_wallichiana_var_cholaensis_XZ_1 | 40 | 8 | 1 | 4 | 0 | 0 | ['A', 'T', 'AT', 'TA', 'AATA', 'ATTT', 'GATA', 'GTCT'] |
| Rhodiola_wallichiana_var_cholaensis_XZ_2 | 39 | 8 | 0 | 4 | 0 | 0 | ['A', 'T', 'AT', 'TA', 'AATA', 'GATA', 'GTCT', 'GTTT'] |
| Rhodiola_wallichiana_var_cholaensis_YN | 38 | 7 | 0 | 4 | 1 | 0 | ['A', 'T', 'AT', 'TA', 'AATA', 'GATA', 'GTCT', 'GTTT'] |
| Rhodiola_wallichiana_XZ | 36 | 9 | 0 | 4 | 0 | 0 | ['A', 'T', 'AT', 'TA', 'TTG', 'AATA', 'GATA', 'GTCT', 'GTTT', 'TATTA'] |
| Rhodiola_yunnanensis_YN_2 | 34 | 7 | 1 | 5 | 0 | 0 | ['A', 'T', 'AT', 'TA', 'AATA', 'ATTT', 'GATA', 'GTCT'] |
| Rhodiola_atuntsuensis_XZ_4 | 41 | 8 | 0 | 4 | 0 | 1 | ['A', 'T', 'AT', 'TA', 'AATA', 'ATTT', 'GATA', 'GTCT'] |
| Rhodiola_discolor_XZ | 34 | 6 | 0 | 4 | 0 | 1 | ['A', 'T', 'AT', 'TA', 'TAA', 'AATA', 'GATA', 'GTCT', 'GTTT'] |
| Rhodiola_yunnanensis_YN_3 | 45 | 7 | 1 | 5 | 0 | 1 | ['A', 'C', 'T', 'AT', 'TA', 'AATA', 'GATA', 'GTCT', 'GTTT'] |
| Rhodiola_himalensis_XZ_1 | 38 | 7 | 0 | 5 | 0 | 2 | ['A', 'T', 'AT', 'TA', 'AATA', 'GATA', 'GTCT', 'GTTT', 'TCTGG'] |
| Rhodiola_kirilowii_XZ_1 | 35 | 7 | 0 | 4 | 0 | 2 | ['A', 'T', 'AT', 'TA', 'AATA', 'GATA', 'GTCT', 'GTTT'] |
| Rhodiola_rosea_JL | 35 | 9 | 1 | 4 | 0 | 2 | ['A', 'T', 'AT', 'TA', 'GTT', 'AATA', 'ATTT', 'GATA', 'GTCT', 'GTTT'] |
| Rhodiola_sacra_YN | 41 | 5 | 0 | 4 | 1 | 2 | ['A', 'T', 'AT', 'TA', 'GTT', 'AATA', 'ATTT', 'GATA', 'GTCT', 'GTTT', 'TTATAG'] |

**Supplementary Table 7. Numbers of tandem repeats and dispersed repeats in *Rhodiola* chloroplast genomes.**

| Genome_name | TRF_num | TRF_len | vmatch-D | vmatch-P |
| --- | --- | --- | --- | --- |
| Rhodiola_algida_QH_2 | 22 | [11, 13, 14, 14, 14, 15, 15, 15, 15, 16, 16, 16, 18, 18, 19, 21, 21, 21, 24, 25, 61, 61] | 9 | 15 |
| Rhodiola_algida_XZ_1 | 20 | [2, 13, 14, 14, 14, 15, 15, 15, 16, 16, 18, 18, 19, 21, 21, 21, 25, 61, 61, 105] | 10 | 14 |
| Rhodiola_algida_XZ_2 | 19 | [11, 13, 13, 14, 14, 14, 15, 15, 15, 16, 16, 18, 18, 19, 21, 21, 21, 61, 61] | 9 | 14 |
| Rhodiola_amabilis_XZ_1 | 19 | [10, 10, 12, 13, 14, 14, 15, 15, 17, 18, 18, 19, 19, 21, 21, 21, 29, 61, 61] | 8 | 14 |
| Rhodiola_amabilis_XZ_2 | 21 | [10, 10, 12, 13, 14, 14, 15, 15, 15, 17, 18, 18, 19, 19, 19, 21, 21, 21, 61, 61, 105] | 8 | 15 |
| Rhodiola_atuntsuensis_XZ_2 | 18 | [13, 14, 14, 14, 15, 15, 15, 16, 16, 18, 18, 19, 21, 21, 21, 25, 61, 61] | 9 | 14 |
| Rhodiola_atuntsuensis_XZ_3 | 21 | [13, 13, 14, 14, 14, 15, 15, 15, 16, 16, 16, 18, 19, 21, 21, 21, 24, 25, 25, 61, 61] | 11 | 14 |
| Rhodiola_bupleuroides_XZ_1 | 22 | [10, 11, 12, 14, 14, 14, 15, 15, 16, 16, 16, 18, 19, 19, 21, 21, 21, 22, 23, 25, 61, 61] | 9 | 14 |
| Rhodiola_bupleuroides_XZ_2 | 16 | [10, 14, 14, 14, 15, 15, 16, 16, 18, 21, 21, 22, 38, 38, 61, 61] | 12 | 16 |
| Rhodiola_chrysanthemifolia_AG | 20 | [2, 10, 10, 12, 13, 14, 14, 14, 15, 15, 15, 18, 18, 19, 19, 21, 21, 21, 61, 61] | 7 | 13 |
| Rhodiola_chrysanthemifolia_XZ_1 | 20 | [2, 10, 10, 12, 13, 14, 14, 14, 15, 15, 15, 18, 18, 19, 19, 21, 21, 21, 61, 61] | 7 | 13 |
| Rhodiola_chrysanthemifolia_XZ_2 | 20 | [2, 10, 10, 12, 13, 14, 14, 14, 15, 15, 15, 18, 18, 19, 19, 21, 21, 21, 61, 61] | 7 | 13 |
| Rhodiola_crenulata_AG | 24 | [2, 10, 13, 13, 14, 14, 14, 15, 15, 16, 16, 16, 18, 20, 20, 21, 21, 21, 22, 22, 25, 31, 61, 61] | 13 | 17 |
| Rhodiola_crenulata_SC | 21 | [2, 10, 13, 13, 14, 14, 14, 15, 15, 16, 16, 16, 18, 20, 21, 21, 22, 24, 25, 61, 61] | 12 | 17 |
| Rhodiola_crenulata_XJ | 22 | [2, 10, 13, 13, 14, 14, 14, 15, 15, 16, 16, 16, 18, 20, 20, 21, 21, 22, 22, 25, 61, 61] | 13 | 17 |
| Rhodiola_crenulata_XZ_1 | 22 | [2, 10, 11, 13, 13, 14, 14, 14, 15, 15, 16, 16, 16, 18, 21, 21, 22, 22, 22, 25, 61, 61] | 19 | 25 |
| Rhodiola_crenulata_XZ_2 | 22 | [2, 10, 11, 13, 13, 14, 14, 14, 15, 15, 16, 16, 16, 18, 21, 21, 22, 22, 22, 25, 61, 61] | 19 | 25 |
| Rhodiola_daochengensis_XZ_1 | 18 | [13, 14, 14, 14, 15, 15, 15, 16, 16, 18, 18, 19, 21, 21, 21, 25, 61, 61] | 9 | 14 |
| Rhodiola_discolor_SC | 20 | [10, 14, 14, 14, 15, 15, 16, 16, 16, 18, 19, 19, 19, 20, 21, 21, 22, 61, 61, 105] | 10 | 12 |
| Rhodiola_dumulosa_NX | 18 | [10, 13, 14, 14, 14, 15, 15, 15, 17, 17, 18, 18, 21, 21, 22, 23, 61, 61] | 7 | 14 |
| Rhodiola_dumulosa_SX_1 | 14 | [10, 12, 13, 14, 14, 14, 15, 15, 18, 21, 21, 22, 61, 61] | 7 | 13 |
| Rhodiola_dumulosa_SX_2 | 15 | [10, 12, 13, 14, 14, 14, 15, 15, 18, 18, 21, 21, 22, 61, 61] | 7 | 13 |
| Rhodiola_fastigiata_AG | 17 | [13, 14, 14, 14, 15, 15, 15, 16, 16, 18, 19, 21, 21, 25, 61, 61, 105] | 10 | 14 |
| Rhodiola_fastigiata_SC_1 | 22 | [10, 10, 13, 14, 14, 14, 15, 15, 16, 16, 16, 16, 17, 18, 21, 21, 22, 23, 27, 28, 61, 61] | 12 | 13 |
| Rhodiola_fastigiata_SC_3 | 20 | [10, 10, 13, 14, 14, 14, 14, 14, 15, 15, 15, 16, 16, 18, 21, 21, 22, 25, 61, 61] | 9 | 14 |
| Rhodiola_fastigiata_XJ_1 | 19 | [2, 11, 13, 14, 14, 14, 15, 15, 15, 16, 18, 18, 19, 21, 21, 21, 25, 61, 61] | 9 | 14 |
| Rhodiola_fastigiata_XJ_2 | 20 | [9, 10, 12, 13, 14, 14, 14, 15, 15, 16, 18, 19, 20, 21, 21, 21, 25, 45, 61, 61] | 11 | 14 |
| Rhodiola_fastigiata_XZ_1 | 19 | [10, 13, 14, 14, 14, 15, 15, 16, 16, 16, 18, 19, 19, 21, 21, 22, 25, 61, 61] | 9 | 14 |
| Rhodiola_fastigiata_XZ_2 | 20 | [13, 14, 14, 14, 14, 15, 15, 15, 16, 16, 18, 18, 19, 21, 21, 21, 25, 61, 61, 105] | 11 | 15 |
| Rhodiola_fastigiata_YN_1 | 22 | [2, 10, 13, 14, 14, 14, 14, 15, 15, 15, 15, 16, 16, 18, 20, 21, 21, 21, 21, 22, 61, 61] | 10 | 18 |
| Rhodiola_forrestii_XZ | 18 | [2, 10, 12, 13, 14, 14, 14, 15, 15, 16, 16, 16, 18, 21, 21, 22, 61, 61] | 10 | 14 |
| Rhodiola_forrestii_YN | 16 | [10, 14, 14, 14, 15, 15, 16, 16, 16, 18, 21, 21, 22, 27, 61, 61] | 10 | 14 |
| Rhodiola_himalensis_XZ_2 | 21 | [9, 13, 13, 14, 14, 14, 15, 15, 15, 16, 16, 16, 18, 19, 20, 21, 21, 24, 29, 61, 61] | 11 | 14 |
| Rhodiola_himalensis_XZ_4 | 21 | [9, 10, 14, 14, 14, 14, 14, 14, 15, 15, 15, 16, 16, 17, 18, 21, 21, 21, 22, 61, 61] | 10 | 17 |
| Rhodiola_hobsonii_XZ | 23 | [10, 10, 12, 13, 13, 14, 14, 15, 15, 15, 18, 18, 18, 19, 19, 19, 20, 21, 21, 21, 21, 61, 61] | 7 | 14 |
| Rhodiola_humilis_var_jialiensis_XZ | 20 | [10, 10, 12, 13, 14, 14, 15, 15, 17, 18, 19, 19, 19, 19, 21, 21, 21, 33, 61, 61] | 8 | 13 |
| Rhodiola_kirilowii_HB | 18 | [2, 12, 14, 14, 14, 15, 15, 15, 16, 16, 18, 19, 21, 21, 21, 25, 61, 61] | 9 | 15 |
| Rhodiola_kirilowii_SX_2 | 22 | [9, 12, 14, 14, 14, 15, 15, 15, 16, 16, 16, 16, 17, 18, 18, 19, 21, 21, 21, 25, 61, 61] | 10 | 14 |
| Rhodiola_kirilowii_XZ_2 | 19 | [13, 13, 14, 14, 14, 14, 15, 15, 16, 16, 16, 18, 19, 19, 21, 21, 25, 61, 61] | 9 | 15 |
| Rhodiola_kirilowii_YN_1 | 19 | [2, 10, 13, 14, 14, 14, 15, 15, 15, 16, 16, 18, 19, 21, 21, 21, 22, 61, 61] | 9 | 14 |
| Rhodiola_kirilowii_YN_2 | 19 | [2, 10, 13, 14, 14, 14, 15, 15, 15, 16, 16, 18, 19, 21, 21, 21, 22, 61, 61] | 9 | 14 |
| Rhodiola_kirilowii_YN_3 | 14 | [10, 11, 14, 14, 14, 15, 15, 16, 16, 16, 18, 21, 21, 22] | 5 | 8 |
| Rhodiola_litwinowii_XJ | 17 | [2, 13, 13, 14, 14, 14, 15, 15, 15, 16, 16, 18, 21, 21, 22, 61, 61] | 9 | 17 |
| Rhodiola_nepalica_XZ_1 | 21 | [10, 10, 12, 13, 14, 14, 14, 15, 15, 17, 18, 18, 19, 19, 19, 21, 21, 21, 39, 61, 61] | 8 | 14 |
| Rhodiola_nepalica_XZ_2 | 21 | [10, 12, 13, 13, 14, 14, 15, 15, 15, 15, 17, 18, 18, 19, 19, 19, 21, 21, 21, 61, 61] | 7 | 13 |
| Rhodiola_nobilis_XZ | 18 | [13, 14, 14, 14, 14, 15, 15, 15, 16, 16, 16, 18, 19, 21, 21, 25, 61, 61] | 9 | 14 |
| Rhodiola_prainii_XZ | 21 | [10, 12, 13, 13, 14, 14, 15, 15, 15, 15, 17, 18, 18, 19, 19, 19, 21, 21, 21, 61, 61] | 7 | 13 |
| Rhodiola_primuloides_YN_1 | 23 | [9, 10, 13, 14, 14, 15, 15, 15, 17, 17, 18, 19, 21, 21, 22, 22, 22, 22, 22, 26, 29, 61, 61] | 9 | 15 |
| Rhodiola_primuloides_YN_2 | 22 | [9, 10, 13, 14, 14, 15, 15, 15, 17, 17, 18, 19, 21, 21, 22, 22, 22, 22, 22, 29, 61, 61] | 8 | 15 |
| Rhodiola_primuloides_YN_3 | 22 | [9, 10, 13, 14, 14, 15, 15, 15, 17, 17, 18, 19, 21, 21, 22, 22, 22, 22, 22, 29, 61, 61] | 8 | 15 |
| Rhodiola_purpureoviridis_SC | 25 | [10, 12, 14, 14, 14, 15, 15, 16, 16, 16, 18, 19, 19, 19, 20, 20, 20, 21, 21, 22, 25, 25, 61, 61, 105] | 11 | 14 |
| Rhodiola_purpureoviridis_XZ_1 | 22 | [10, 14, 14, 14, 15, 15, 16, 16, 17, 17, 18, 19, 19, 21, 21, 22, 22, 22, 38, 38, 61, 61] | 12 | 16 |
| Rhodiola_purpureoviridis_XZ_2 | 25 | [1, 9, 10, 14, 14, 14, 15, 15, 16, 16, 18, 18, 19, 19, 19, 19, 21, 21, 21, 22, 23, 25, 36, 61, 61] | 11 | 14 |
| Rhodiola_quadrifida_XZ_1 | 20 | [10, 10, 14, 14, 14, 15, 15, 16, 16, 16, 16, 18, 18, 19, 19, 21, 21, 61, 61, 105] | 11 | 15 |
| Rhodiola_quadrifida_XZ_2 | 20 | [10, 10, 14, 14, 14, 15, 15, 16, 16, 16, 16, 18, 18, 19, 19, 21, 21, 61, 61, 105] | 11 | 16 |
| Rhodiola_quadrifida_XZ_3 | 17 | [2, 10, 11, 13, 14, 14, 15, 15, 15, 16, 17, 18, 21, 21, 23, 61, 61] | 12 | 19 |
| Rhodiola_rosea_HB_1 | 29 | [7, 10, 12, 13, 14, 15, 15, 16, 16, 18, 18, 19, 19, 19, 20, 20, 21, 21, 22, 22, 22, 22, 26, 27, 38, 61, 61, 65, 102] | 53 | 13 |
| Rhodiola_rosea_HB_2 | 25 | [2, 9, 10, 11, 13, 13, 14, 14, 15, 15, 15, 16, 16, 17, 17, 18, 19, 21, 21, 22, 25, 31, 44, 61, 61] | 9 | 15 |
| Rhodiola_rosea_HB_3 | 25 | [2, 9, 10, 11, 13, 13, 14, 14, 15, 15, 15, 16, 16, 17, 17, 18, 19, 21, 21, 22, 25, 31, 44, 61, 61] | 9 | 15 |
| Rhodiola_rosea_HB_4 | 20 | [10, 12, 13, 14, 15, 15, 15, 16, 16, 18, 18, 19, 19, 21, 21, 22, 22, 22, 61, 61] | 11 | 12 |
| Rhodiola_rosea_XJ_1 | 16 | [10, 13, 14, 15, 15, 16, 16, 18, 19, 19, 20, 21, 21, 22, 61, 61] | 8 | 13 |
| Rhodiola_rosea_XJ_2 | 23 | [10, 13, 14, 14, 14, 14, 15, 15, 16, 16, 17, 18, 19, 19, 19, 19, 20, 21, 21, 22, 24, 61, 61] | 9 | 13 |
| Rhodiola_sachalinensis_JL_1 | 26 | [2, 10, 12, 13, 13, 13, 14, 14, 15, 15, 17, 18, 18, 20, 20, 21, 21, 21, 22, 22, 22, 23, 24, 27, 61, 61] | 11 | 13 |
| Rhodiola_sacra_var_tsuiana_XZ | 20 | [10, 10, 12, 13, 13, 14, 14, 15, 15, 16, 16, 18, 19, 19, 19, 21, 21, 21, 61, 61] | 7 | 13 |
| Rhodiola_sacra_XZ_1 | 23 | [10, 10, 12, 13, 13, 14, 14, 15, 15, 15, 18, 18, 18, 19, 19, 19, 20, 21, 21, 21, 21, 61, 61] | 7 | 14 |
| Rhodiola_sacra_XZ_2 | 22 | [10, 12, 13, 13, 14, 14, 15, 15, 15, 15, 17, 18, 18, 19, 19, 19, 19, 21, 21, 21, 61, 61] | 9 | 15 |
| Rhodiola_sacra_XZ_3 | 24 | [11, 12, 12, 13, 13, 14, 14, 14, 15, 15, 16, 17, 17, 18, 18, 19, 19, 19, 19, 21, 21, 23, 61, 61] | 8 | 14 |
| Rhodiola_sacra_XZ_4 | 22 | [10, 10, 12, 13, 13, 14, 14, 15, 15, 15, 17, 17, 18, 18, 19, 19, 21, 21, 21, 24, 61, 61] | 8 | 14 |
| Rhodiola_sacra_XZ_5 | 21 | [10, 10, 12, 13, 14, 14, 14, 15, 15, 17, 18, 18, 19, 19, 19, 21, 21, 21, 24, 61, 61] | 8 | 14 |
| Rhodiola_serrata_XZ_1 | 18 | [10, 14, 14, 14, 14, 15, 15, 15, 16, 16, 18, 21, 21, 22, 38, 38, 61, 61] | 12 | 18 |
| Rhodiola_serrata_XZ_2 | 19 | [10, 14, 14, 14, 15, 15, 16, 16, 17, 18, 19, 19, 21, 21, 22, 38, 38, 61, 61] | 12 | 17 |
| Rhodiola_sexifolia_XZ | 20 | [10, 10, 12, 13, 13, 14, 14, 15, 15, 16, 18, 18, 19, 19, 19, 21, 21, 21, 61, 61] | 7 | 13 |
| Rhodiola_subopposita_XZ | 22 | [9, 10, 10, 13, 14, 14, 14, 15, 15, 16, 16, 17, 18, 19, 19, 19, 21, 21, 21, 25, 61, 61] | 10 | 14 |
| Rhodiola_tangutica_XZ_2 | 19 | [11, 13, 14, 14, 14, 15, 15, 15, 16, 16, 18, 18, 19, 21, 21, 21, 25, 61, 61] | 9 | 14 |
| Rhodiola_tibetica_XZ | 19 | [10, 13, 14, 14, 14, 15, 15, 16, 16, 16, 18, 19, 21, 21, 21, 24, 24, 61, 61] | 9 | 13 |
| Rhodiola_tieghemii_XZ_1 | 20 | [10, 10, 10, 12, 13, 13, 14, 14, 15, 15, 18, 18, 18, 19, 19, 21, 21, 21, 61, 61] | 7 | 14 |
| Rhodiola_tieghemii_XZ_2 | 25 | [10, 11, 12, 13, 13, 14, 14, 14, 15, 15, 18, 18, 18, 19, 19, 19, 19, 20, 21, 21, 23, 38, 38, 61, 61] | 12 | 17 |
| Rhodiola_wallichiana_var_cholaensis_XZ_1 | 23 | [11, 13, 13, 13, 14, 14, 14, 15, 15, 15, 16, 16, 16, 16, 18, 19, 21, 21, 21, 25, 28, 71, 71] | 8 | 12 |
| Rhodiola_wallichiana_var_cholaensis_XZ_2 | 19 | [2, 10, 14, 14, 15, 15, 16, 16, 16, 18, 18, 19, 21, 21, 23, 25, 25, 61, 61] | 9 | 15 |
| Rhodiola_wallichiana_var_cholaensis_YN | 19 | [2, 10, 13, 14, 14, 14, 15, 15, 15, 16, 16, 18, 19, 21, 21, 21, 22, 61, 61] | 9 | 14 |
| Rhodiola_wallichiana_XZ | 18 | [11, 13, 14, 14, 14, 15, 15, 15, 16, 16, 18, 18, 19, 21, 21, 25, 61, 61] | 9 | 14 |
| Rhodiola_yunnanensis_YN_2 | 14 | [10, 14, 14, 14, 15, 15, 16, 16, 18, 21, 21, 22, 61, 61] | 9 | 13 |
| Rhodiola_atuntsuensis_XZ_4 | 19 | [10, 13, 14, 14, 14, 15, 15, 15, 16, 16, 16, 18, 19, 20, 21, 21, 21, 61, 61] | 9 | 14 |
| Rhodiola_discolor_XZ | 19 | [10, 14, 14, 14, 15, 15, 16, 16, 17, 18, 19, 19, 21, 21, 21, 22, 38, 38, 105] | 9 | 12 |
| Rhodiola_yunnanensis_YN_3 | 14 | [10, 10, 14, 14, 14, 15, 15, 16, 16, 16, 18, 21, 21, 22] | 5 | 11 |
| Rhodiola_himalensis_XZ_1 | 20 | [10, 10, 10, 14, 14, 14, 15, 15, 16, 16, 16, 18, 18, 19, 19, 21, 21, 22, 61, 61] | 9 | 15 |
| Rhodiola_kirilowii_XZ_1 | 18 | [10, 10, 13, 14, 14, 14, 15, 15, 16, 16, 18, 18, 19, 21, 21, 25, 61, 61] | 11 | 15 |
| Rhodiola_rosea_JL | 19 | [10, 14, 15, 15, 15, 16, 16, 17, 18, 18, 19, 19, 19, 21, 21, 22, 23, 61, 61] | 11 | 15 |
| Rhodiola_sacra_YN | 22 | [10, 10, 12, 13, 14, 14, 14, 15, 15, 16, 17, 17, 18, 19, 19, 19, 19, 21, 21, 21, 61, 61] | 8 | 13 |

**Supplementary Table 8. Pi values of protein-coding genes (PCGs) and intergenic spacers (IGSs) in *Rhodiola* chloroplast genomes.**

| Gene name | Pi | Intergenic spacer | Pi | Intergenic spacer | Pi |
| --- | --- | --- | --- | --- | --- |
| *petN* | 0 | *psaB+psaA* | 0 | *rps14+psaB* | 0.007806 |
| *rpl2* | 0.000128 | *psbL+psbF-merge-psbE* | 0 | *petN+psbM* | 0.007853 |
| *rpl23* | 0.000276 | *trnI-CAU+ycf2* | 0 | *trnS-UGA+psbZ* | 0.007857 |
| *psbL* | 0.000301 | *ycf2_copy1+trnI-CAU_copy1* | 0 | *rpl33+rps18* | 0.007982 |
| *psbJ* | 0.000345 | *rrn16+trnI-GAU* | 0.000125 | *atpF+atpH* | 0.007983 |
| *rpl36* | 0.000347 | *ndhB_copy1+trnL-CAA_copy1* | 0.000131 | *ndhK+ndhC* | 0.008272 |
| *psbF* | 0.000394 | *trnV-GAC+rrn16* | 0.00015 | *psbM+trnD-GUC* | 0.008395 |
| *ndhB* | 0.000396 | *rrn16_copy1+trnV-GAC_copy1* | 0.00015 | *psbI+trnS-GCU* | 0.008597 |
| *psaC* | 0.000396 | *trnI-GAU_copy1+rrn16_copy1* | 0.000192 | *rps12_part1+clpP* | 0.008885 |
| *rpl14* | 0.000449 | *ndhB+rps7* | 0.000218 | *trnP-UGG+psaJ* | 0.009224 |
| *psbE* | 0.000517 | *rps7_copy1+ndhB_copy1* | 0.000218 | *psbH+petB* | 0.009265 |
| *rps14* | 0.000679 | *ycf1+trnN-GUU_copy1* | 0.000275 | *trnT-GGU+psbD-merge-psbC* | 0.009364 |
| *rps7* | 0.001078 | *rps19+rpl2* | 0.000361 | *rps8+rpl14* | 0.009492 |
| *rps12* | 0.001191 | *trnA-UGC_copy1+trnI-GAU_copy1* | 0.000374 | *trnS-GGA+rps4* | 0.009684 |
| *psbD* | 0.001196 | *rpl23+trnI-CAU* | 0.000424 | *rps16+trnQ-UUG* | 0.009734 |
| *psbT* | 0.001212 | *trnI-CAU_copy1+rpl23_copy1* | 0.000424 | *rbcL+accD* | 0.010458 |
| *rps4* | 0.001237 | *trnL-CAA+ndhB* | 0.000549 | *trnE-UUC+trnT-GGU* | 0.010551 |
| *ycf2* | 0.001322 | *trnI-GAU+trnA-UGC* | 0.000663 | *ndhA-merge-ndhH+rps15* | 0.010789 |
| *atpE* | 0.001345 | *rpl36+infA* | 0.001084 | *petA+psbJ* | 0.010877 |
| *ndhK* | 0.001349 | *psbT+psbN* | 0.001229 | *atpI+rps2* | 0.010957 |
| *psbC* | 0.001352 | *trnN-GUU+ndhF* | 0.001278 | *trnK-UUU-merge-matK+rps16* | 0.011084 |
| *petL* | 0.001359 | *psbD-merge-psbC+trnS-UGA* | 0.001511 | *rpl16+rps3* | 0.011183 |
| *psbB* | 0.001363 | *rrn4.5+rrn5* | 0.001643 | *rpoC2+rpoC1* | 0.011197 |
| *rps2* | 0.001565 | *rrn5_copy1+rrn4.5_copy1* | 0.001643 | *ndhI+ndhA-merge-ndhH* | 0.011474 |
| *atpB* | 0.001654 | *atpA+atpF* | 0.001667 | *rpl22+rps19* | 0.011491 |
| *psaA* | 0.001826 | *ycf2+trnL-CAA* | 0.001688 | *petG+trnW-CCA* | 0.012236 |
| *petB* | 0.00184 | *trnL-CAA_copy1+ycf2_copy1* | 0.001688 | *trnS-GCU+trnG-GCC* | 0.012444 |
| *petG* | 0.001886 | *rps12_part2+trnV-GAC* | 0.001767 | *trnQ-UUG+psbK* | 0.012763 |
| *psaB* | 0.002002 | *trnV-GAC_copy1+rps12_part2_copy1* | 0.001767 | *psbB+psbT* | 0.012805 |
| *rbcL* | 0.00206 | *rpl2+rpl23* | 0.001811 | *ndhG+ndhI* | 0.012894 |
| *psbM* | 0.002183 | *rpl23_copy1+rpl2_copy1* | 0.001811 | *trnC-GCA+petN* | 0.013067 |
| *ndhC* | 0.002187 | *trnN-GUU_copy1+trnR-ACG_copy1* | 0.001976 | *psbA+trnK-UUU-merge-matK* | 0.013112 |
| *rps19* | 0.0024 | *trnR-ACG+trnN-GUU* | 0.00202 | *trnL-UAG+ccsA* | 0.013246 |
| *ndhA* | 0.002421 | *rrn5+trnR-ACG* | 0.002037 | *rpoB+trnC-GCA* | 0.013353 |
| *psbH* | 0.002447 | *trnY-GUA+trnE-UUC* | 0.00214 | *trnW-CCA+trnP-UGG* | 0.014138 |
| *petD* | 0.002503 | *trnfM-CAU+rps14* | 0.00233 | *rps15+ycf1* | 0.01424 |
| *infA* | 0.002572 | *rpoA+rps11* | 0.002681 | *rps3+rpl22* | 0.014675 |
| *psbK* | 0.002668 | *rpl2_copy1+trnH-GUG* | 0.002757 | *psbF-merge-psbE+petL* | 0.014736 |
| *rpoC1* | 0.002809 | *trnR-ACG_copy1+rrn5_copy1* | 0.003198 | *trnD-GUC+trnY-GUA* | 0.015011 |
| *atpA* | 0.003012 | *rps7+rps12_part2* | 0.003266 | *trnG-GCC+trnR-UCU* | 0.015522 |
| *psaJ* | 0.003059 | *rps12_part2_copy1+rps7_copy1* | 0.003266 | *ndhF+rpl32* | 0.015595 |
| *atpH* | 0.003074 | *petL+petG* | 0.003549 | *rpoC1+rpoB* | 0.018094 |
| *atpI* | 0.003187 | *trnV-UAC+trnM-CAU* | 0.003612 | *ccsA+ndhD* | 0.018199 |
| *rpoB* | 0.003228 | *trnG-UCC+trnfM-CAU* | 0.003852 | *petB+petD* | 0.018452 |
| *rps11* | 0.003449 | *petD+rpoA* | 0.003962 | *rpl14+rpl16* | 0.02129 |
| *accD* | 0.003486 | *clpP+psbB* | 0.004054 | *trnH-GUG+psbA* | 0.032942 |
| *rpl32* | 0.003765 | *cemA+petA* | 0.00412 | *trnR-UCU+atpA* | 0.034164 |
| *rpl16* | 0.003834 | *psbN+psbH* | 0.004189 |  |  |
| *ndhJ* | 0.004163 | *infA+rps8* | 0.004425 |  |  |
| *rpoC2* | 0.00422 | *rpl20+rps12_part1* | 0.004597 |  |  |
| *ccsA* | 0.004224 | *rps4+trnT-UGU* | 0.004864 |  |  |
| *ycf3* | 0.004437 | *psaA+ycf3* | 0.004927 |  |  |
| *clpP* | 0.004451 | *atpH+atpI* | 0.005069 |  |  |
| *rpl22* | 0.004475 | *ndhJ+ndhK* | 0.00549 |  |  |
| *psbA* | 0.004515 | *trnL-UAA+trnF-GAA* | 0.005697 |  |  |
| *ycf4* | 0.004555 | *trnM-CAU+atpE-merge-atpB* | 0.005811 |  |  |
| *ndhE* | 0.004819 | *psaJ+rpl33* | 0.005965 |  |  |
| *ndhH* | 0.004828 | *trnT-UGU+trnL-UAA* | 0.006089 |  |  |
| *ndhG* | 0.004906 | *psbJ+psbL* | 0.006125 |  |  |
| *atpF* | 0.005064 | *ycf3+trnS-GGA* | 0.006188 |  |  |
| *psbZ* | 0.005199 | *rpl32+trnL-UAG* | 0.006274 |  |  |
| *ndhD* | 0.005273 | *trnA-UGC+rrn23* | 0.006455 |  |  |
| *petA* | 0.005453 | *rrn23_copy1+trnA-UGC_copy1* | 0.006455 |  |  |
| *rps16* | 0.005502 | *ndhC+trnV-UAC* | 0.006492 |  |  |
| *cemA* | 0.005722 | *rps18+rpl20* | 0.006731 |  |  |
| *rpl33* | 0.005999 | *ndhE+ndhG* | 0.006781 |  |  |
| *psbN* | 0.006225 | *psbZ+trnG-UCC* | 0.006835 |  |  |
| *rps8* | 0.006762 | *trnF-GAA+ndhJ* | 0.006934 |  |  |
| *rpoA* | 0.006895 | *rps11+rpl36* | 0.007001 |  |  |
| *rps18* | 0.007359 | *psaC+ndhE* | 0.007151 |  |  |
| *psaI* | 0.007408 | *ndhD+psaC* | 0.007194 |  |  |
| *psbI* | 0.00776 | *psaI+ycf4* | 0.007323 |  |  |
| *ndhF* | 0.008075 | *ycf4+cemA* | 0.007382 |  |  |
| *rps3* | 0.008248 | *accD+psaI* | 0.007585 |  |  |
| *ndhI* | 0.008697 | *psbK+psbI* | 0.007607 |  |  |
| *rpl20* | 0.008763 | *rps2+rpoC2* | 0.007625 |  |  |
| *matK* | 0.009085 | *rrn23+rrn4.5* | 0.0077 |  |  |
| *ycf1* | 0.009606 | *rrn4.5_copy1+rrn23_copy1* | 0.0077 |  |  |
| *rps15* | 0.011398 | *atpE-merge-atpB+rbcL* | 0.007754 |  |  |

**Supplementary Table 9. Numbers of SNP sites in PCGs of Rhodiola chloroplast genomes.**

| Genes | SNPs | Genes | SNPs | Genes | SNPs |
| --- | --- | --- | --- | --- | --- |
| *accD* | 76 | *petL* | 5 | *rpl22* | 34 |
| *atpA* | 53 | *petN* | 0 | *rpl23* | 2 |
| *atpB* | 34 | *psaA* | 66 | *rpl32* | 11 |
| *atpE* | 19 | *psaB* | 50 | *rpl33* | 11 |
| *atpF* | 25 | *psaC* | 6 | *rpl36* | 4 |
| *atpH* | 3 | *psaI* | 2 | *rpoA* | 57 |
| *atpI* | 20 | *psaJ* | 7 | *rpoB* | 115 |
| *ccsA* | 54 | *psbA* | 53 | *rpoC1* | 72 |
| *cemA* | 55 | *psbB* | 37 | *rpoC2* | 203 |
| *clpP* | 19 | *psbC* | 25 | *rps11* | 18 |
| *infA* | 14 | *psbD* | 19 | *rps12* | 7 |
| *matK* | 122 | *psbE* | 6 | *rps14* | 14 |
| *ndhA* | 44 | *psbF* | 2 | *rps15* | 20 |
| *ndhB* | 18 | *psbH* | 9 | *rps16* | 44 |
| *ndhC* | 17 | *psbI* | 7 | *rps18* | 16 |
| *ndhD* | 78 | *psbJ* | 4 | *rps19* | 25 |
| *ndhE* | 9 | *psbK* | 13 | *rps2* | 27 |
| *ndhF* | 216 | *psbL* | 4 | *rps3* | 42 |
| *ndhG* | 35 | *psbM* | 5 | *rps4* | 15 |
| *ndhH* | 64 | *psbN* | 8 | *rps7* | 7 |
| *ndhI* | 24 | *psbT* | 5 | *rps8* | 32 |
| *ndhJ* | 21 | *psbZ* | 7 | *ycf1* | 460 |
| *ndhK* | 17 | *rbcL* | 30 | *ycf2* | 79 |
| *petA* | 64 | *rpl14* | 7 | *ycf3* | 13 |
| *petB* | 12 | *rpl16* | 21 | *ycf4* | 27 |
| *petD* | 9 | *rpl2* | 17 |  |  |
| *petG* | 5 | *rpl20* | 24 |  |  |
